# Five new polyketides from the basidiomycete *Craterellus odoratus*

**DOI:** 10.1007/s13659-012-0057-5

**Published:** 2012-08-07

**Authors:** Hua Guo, Tao Feng, Zheng-Hui Li, Ji-Kai Liu

**Affiliations:** 1 State Key Laboratory of Phytochemistry and Plant Resources in West China, Kunming Institute of Botany, Chinese Academy of Sciences, Kunming, 650201 China; 2 School of Chemistry and Life Science, Anshan Normal College, Anshan, 114005 China; 3 Graduate University of Chinese Academy of Sciences, Beijing, 100049 China

**Keywords:** *Craterellus odoratus*, craterellones, polyketides

## Abstract

Five new polyketides, craterellones A–E (**1–5**), were isolated from cultures of basidiomycete *Craterellus odoratus*, together with five known compounds (**6–10**). Structures of **1–5** were elucidated on the basis of extensive spectroscopic analysis. All compounds were evaluated for their inhibitory activities against one isozyme of 11*β*-hydroxysteroid dehydrogenase (11*β*-HSD1) and cytotoxic activities on five tumor cell lines. Compound **10** exhibited significant cytotoxicity against HL-60, SMMC-7721, A-549, MCF-7, and SW-480, with IC50 values of 0.50, 0.69, 0.64, 1.10, 0.54 µM, respectively.

**Electronic Supplementary Material:**

Supplementary material is available for this article at 10.1007/s13659-012-0057-5 and is accessible for authorized users.

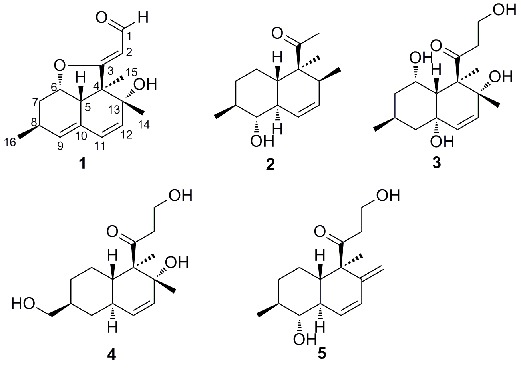

## Introduction

*Craterellus odoratus* (Schwein.) Fr. (Cantharellaceae) is an edible fungus, which is widespread in mainland China and characterized by possessing a bright orange or yellow cap. Our previous study on the secondary metabolites of *C. odoratus* has reported a series of merosesquiterpenoids, while one of them demonstrated significant inhibitory activities against human 11*β*-HSD2.[1] As a part of our continuous search for further new bioactive constituents, an enlarged culture on the same resource was investigated, which resulted in the isolation of five new compounds, craterellones A–E (**1**–**5**), together with five known compounds. The structures of the new compounds were established on the basis of extensive spectroscopic methods, while the known compounds were identified as decumbenones A and B (**6** and **7**),[2] versiol (**8**),[3] calbistrin A (**9**),[4] and calbistrin C (**10**),[4] respectively, by comparison with data as reported in the literature. All compounds were evaluated for their cytotoxicities against five human cancer lines and their inhibitory activities against one isozyme of 11*β*-hydroxysteroid dehydrogenase (11*β*-HSD1). This paper deals with the isolation, structural elucidation, and bioactivities of these isolates.

## Results and Discussion

Craterellone A (**1**) was obtained as white powder. Its molecular formula was determined to be $C_{16}H_{20}O_3$ by HRESIMS, corresponding to seven degrees of unsaturation.

The IR spectrum showed absorption bands for a hydroxy group (3431 $cm^{-1}$) and double bonds (1635 $cm^{-1}$). The $^{13}C$ NMR and DEPT spectra showed 16 carbon signals that attributed to three methyls, one methylene, eight methines, and four quaternary carbons. Comparison of the NMR data of **1** with those of decumbenone A (**6**)[2] revealed that compound **1** was a polyketide. The $^{13}C$ NMR data indicated that the carbon signals corresponding to two $sp^3$ methylenes and a carbonyl group ($\delta_C$ 215.0) in **6** were not present in **1**. Instead, one $sp^2$ quaternary carbon at $\delta_C$ 181.3 (s, C-3), one $sp^2$ methine at $\delta_C$ 102.8 (d, C-2), and an aldehyde carbon at $\delta_C$ 188.9 (d, C-1) were observed. The $^1H$-$^1H$ COSY correlation of H-1/H-2 and the HMBC correlations (Figure 1) of H-1/C-2 ($\delta_C$ 102.8), and H-2/C-3 ($\delta_C$ 181.3) and C-4 ($\delta_C$ 53.6) concluded C-1, C-2 and C-3 as an *α*,*β*-unsaturated aldehyde group. In addition to six

*To whom correspondence should be addressed. E-mail: jkliu@mail.kib.ac.cn

Springer

**Table 1. $^1$H NMR spectroscopic data (400 MHz) of craterellones A–E (1–5)**

| position | 1[a] | 2[a] | 3[b] | 4[a] | 5[a] |
|---|---|---|---|---|---|
| 1a | 10.49, d (8.6) | | 3.81, m | 4.29, m | 4.29, br. s |
| 1b | | | | | |
| 2a | 5.92, d (8.6) | 2.09, s | 3.10, m | 3.64, m | 3.16, m |
| 2b | | | 2.80, m | 3.05, m | 2.83, m |
| 5 | 3.12, d (9.1) | 1.94, m | 2.05, m | 2.17, m | 2.03, m |
| 6a | 4.91, m | 1.83, m | 4.24, m | 1.94, m | 1.32, m |
| 6b | | 1.00, m | | 1.08, m | 1.25, m |
| 7a | 1.97, m | 1.74, m | 1.86, m | 2.01, m | 1.67, m |
| 7b | 1.86, m | 1.22, m | 1.28, m | 1.28, m | 1.04, m |
| 8 | 2.52, m | 1.63, m | 2.33, m | 1.79, m | 1.61, m |
| 9a | 5.77, d (5.9) | 3.12, dd (??) | 1.78, m | 2.08, m | 3.07, m |
| 9b | | | 1.08, m | 1.03, m | |
| 10 | | 1.95, m | | 1.86, m | 2.16, m |
| 11 | 6.14, d (9.6) | 6.52, d (10.0) | 5.42, d (9.9) | 5.42, d (10.0) | 6.75, d (9.6) |
| 12 | 5.73, d (9.6) | 5.67, m | 5.43, d (9.9) | 5,71, d (10.0) | 6.33, d (9.6) |
| 13 | | 2.05, m | | | |
| 14a | 1.71, s | 0.84,d (7.2) | 1.03, s | 1.49, s | 4.99, s |
| 14b | | | | | 4.88, s |
| 15 | 1.49, s | 1.18, s | 1.82, s | 1.55, s | 1.28, s |
| 16 | 1.00, d (7.4) | 1.22, d (6.3) | 0.96, d (6.6) | 3.70, d (5.9) | 1.21, d (6.2) |
| OH-10 | | | 5.29, s | | |

[a]Measured in pyridine-$d_5$; [b]Measured in acetone-$d_6$.

degrees of unsaturation occupied by two rings, three double bonds, and one aldehyde, the remaining one degree of unsaturation required that compound **1** had an ether ring in the form of a 3,6-ether moiety, which was in agreement with the significant downfield signals for C-3 ($\delta_C$ 181.3) and C-6 ($\delta_C$ 79.3) and the HMBC correlation from H-6 to C-3. The relative configuration of **1** was assigned on basis of the ROESY experiment (Figure 1). ROESY correlations of Me-14 with H-2 and H-5, of Me-16 with H-6 and H-7$\beta$, and of Me-15 with H-7$\alpha$ indicated that **1** had the same configuration as that of **6**. Therefore, the structure of **1** was determined as shown, and named as craterellone A.

**Figure 1.** Key $^1$H-$^1$H COSY, HMBC and ROESY correlations of **1**

Craterellone B (**2**) exhibited the molecular formula $C_{15}H_{24}O_2$, as determined by its HRESIMS at *m/z* 259.1678 ([M + Na]$^+$), corresponding to four degrees of unsaturation. Comparison of NMR data suggested that **2** still possessed a polyketide skeleton related to that of **1**. Analysis of the $^1$H-$^1$H COSY spectrum revealed a partial structure, giving rise to the same bicyclic system to that of **1**. Continuous analysis of HMBC spectrum revealed the main difference to be the length of the side chain of carbons at C-1, C-2, and C-3. Carbon resonances at $\delta_C$ 27.5 (q, C-2) and $\delta_C$ 211.4 (s, C-3) are typical for an acetyl group, which was suggested to be connected to C-4 by the HMBC correlations of H-2 to C-4. Analysis of other 2D NMR data established compound **2** to be a 1-nor-polyketide of **1**. According to the ROESY experiment, compound **2** was found to possess the same relative configuration with that of **1**. In addition, the ROESY correlation of H-9 with H-5 and Me-16 suggested the $\alpha$-orientation of OH-9, while the ROESY correlation of H-10 with H-15 suggested the $\alpha$-orientation of H-10. Thus, the structure of **2** (craterellone B) was established as shown.

Craterellone C (**3**) was isolated as a yellow oil and found to possess a molecular formula of $C_{16}H_{26}O_5$, as deduced from its HRESIMS at *m/z* 321.1668 [M + Na]$^+$. The $^1$H and $^{13}$C NMR spectra of **3** were similar to those of **7**.[2] Careful comparison of their NMR data indicated that **3** was a hydroxy derivative of **7**, as explained by the oxygenated quaternary carbon at $\delta_C$ 71.5. This quaternary carbon at $\delta_C$ 71.5 (s) was assigned to C-10 according to the HMBC correlations from H-5, H-9, and H-11 to C-10. The ROESY correlation of OH-10 with Me-15 implied the OH-10 to be $\alpha$-oriented. Consequently, the structure of craterellone C was proposed as **3**.

Craterellone D (**4**) was obtained as yellow, amorphous powder. Its molecular formula was assigned to be $C_{16}H_{26}O_4$, the same to that of **7**. Comparison of the spectroscopic data of **4** with those of **7** also indicated the similar patterns except for signals of an oxygenated methylene [$\delta_H$ 3.70 (2H, d, $J$ = 5.9 Hz, H-16); $\delta_C$ 67.8 (t, C-16)] in **4** instead of those of the oxygenated methine in **7**. Analysis of $^1$H-$^1$H COSY and HMBC spectra revealed the hydroxy substitution at C-16. Detailed analysis of other spectroscopic data (HSQC, HMBC, $^1$H-$^1$H COSY, ROESY) established the structure of compound **4** (craterellone D) as shown.

The molecular formula of craterellone E (**5**) was inferred to be $C_{16}H_{20}O_3$ on the basis of its positive HRESIMS. The IR spectrum indicated the presence of hydroxy (3431 cm$^{-1}$), carbonyl group (1701 cm$^{-1}$), and conjugated terminal double bonds (1630 cm$^{-1}$). Preliminary analysis of the NMR data indicated that **5** possessed the same skeleton to that of **7**. HMBC correlations of $\delta_H$ 3.07 (1H, m, H-9) with C-8 and C-10 indicated the OH substitution at C-9. In addition, $^1$H NMR signals of the terminal double bonds at $\delta_H$ 4.99 and 4.88 (each 1H, s, H-14) showed the HMBC correlations to C-13, C-4, and C-12, suggesting that the terminal double bond was constructed at C-13 and C-14. The ROESY correlation of H-9 with H-5 indicated H-9 to be $\beta$-oriented, the same to that of **2**. Thus, compound **5** (craterellone E) was established as shown.

**Table 2.** $^{13}C$ **NMR spectroscopic data (100 MHz) of craterellones A–E (1–5)**

| pos. | **1**[a] | **2**[a] | **3**[b] | **4**[a] | **5**[a] |
|---|---|---|---|---|---|
| 1 | 188.9, CH | | 57.9, $CH_2$ | 57.8, $CH_2$ | 58.1, $CH_2$ |
| 2 | 102.8, CH | 27.5, $CH_3$ | 44.8, $CH_2$ | 45.7, $CH_2$ | 41.4, $CH_2$ |
| 3 | 181.3, qC | 211.4, qC | 215.1, qC | 214.3, qC | 211.7, qC |
| 4 | 53.6, qC | 52.5, qC | 57.7, qC | 57.9, qC | 56.7, qC |
| 5 | 41.9, CH | 37.4, CH | 44.5, CH | 44.3, CH | 42.8, CH |
| 6 | 79.3, CH | 27.3, $CH_2$ | 69.8, CH | 27.6, $CH_2$ | 26.7, $CH_2$ |
| 7 | 32.5, $CH_2$ | 34.1, $CH_2$ | 44.4, $CH_2$ | 30.5, $CH_2$ | 33.3, $CH_2$ |
| 8 | 31.4, CH | 41.7, CH | 23.0, CH | 42.0, CH | 41.0, CH |
| 9 | 127.7, CH | 78.9, CH | 49.9, $CH_2$ | 36.9, $CH_2$ | 78.3, CH |
| 10 | 131.8, qC | 46.3, CH | 71.5, qC | 38.6, CH | 44.6, CH |
| 11 | 126.6, CH | 126.7, CH | 131.1, CH | 129.7, CH | 131.8, CH |
| 12 | 139.6, CH | 130.4, CH | 135.1, CH | 135.6, CH | 128.5, CH |
| 13 | 75.1, qC | 39.4, CH | 74.1, qC | 73.3, qC | 148.3, qC |
| 14 | 24.1, $CH_3$ | 18.9, $CH_3$ | 26.9, $CH_3$ | 28.6, $CH_3$ | 112.7, $CH_2$ |
| 15 | 20.7, $CH_3$ | 17.8, $CH_3$ | 15.1, $CH_3$ | 12.7, $CH_3$ | 17.9, $CH_3$ |
| 16 | 20.4, $CH_3$ | 19.5, $CH_3$ | 22.0, $CH_3$ | 67.8, $CH_2$ | 19.3, $CH_3$ |

[a]Measured in pyridine-$d_5$; [b]Measured in acetone-$d_6$.

All compounds were evaluated for their inhibitory activities against one isozyme of 11$\beta$-hydroxysteroid dehydrogenase (11$\beta$-HSD1) and their cytotoxicities against five human cancer cell lines. The results showed that only compound **10** exhibited significant cytotoxicity against HL-60, SMMC-7721, A-549, MCF-7, and SW-480, with $IC_{50}$ values of 0.50, 0.69, 0.64, 1.10, 0.54 $\mu$M, respectively.

## Experimental Section

**General Experimental Procedures.** Optical rotations were measured on a Jasco-P-1020 polarimeter. UV spectra were measured on a Shimadzu UV-2401 PC spectrophotometer. IR spectra were obtained by using a Bruker Tensor 27 FT-IR spectrometer with KBr pellets. NMR spectra were acquired with instruments of Bruker DRX-500 or Bruker AV 400. ESIMS and HRESIMS were measured on Bruker HCT/Esquire and API QSTAR Pulsar, respectively. Preparative HPLC was performed on an Agilent 1100 series with a Zorbax SB-C18 (5 $\mu$m, 9.4 × 150 mm) column. Preparative MPLC was performed on a Büchi apparatus equipped with Büchi fraction collector C-660, Büchi pump module C-605 and manager C-615. Silica gel (200–300 mesh and 80–100 mesh, Qingdao Marine Chemical Inc., China), RP-18 gel (40–75 $\mu$m, Fuji Silysia Chemical Ltd., Japan) and Sephadex LH-20 (Amersham Biosciences, Sweden) were used for column chromatography. Fractions were monitored by TLC (Qingdao Marine Chemical Inc., China) and spots visualized by heating silica gel plates immersed in vanillin-$H_2SO_4$ in EtOH.

**Fungal Material and Cultivation Conditions.** The fungus *C. odoratus* was collected from the southern part of the Gaoligong Mountains in Yunnan Province, China, in July 2007. The fungus was identified by Prof. Mu Zang at the Kunming Institute of Botany. A voucher specimen (HFG07004) was deposited at the Herbarium of Kunming Institute of Botany. Culture medium: glucose (5%), pork peptone (0.15%), yeast (0.5%), $KH_2PO_4$ (0.05%), $MgSO_4$ (0.05%), The initial pH was adjusted to 6.0, the fermentation was first carried out on an erlenmeyer flask for six days till the mycelium biomass reached to the maximum. Later it was transferred to a fermentation tank (100 L) at 24 °C and 250 rpm for twenty days, ventilation was settled to 1.0 vvm (vvm: air volume/culture volumn/min).

**Extraction and Isolation.** The culture broth (70 L) was extracted three times with EtOAc (3 × 10 L). The combined EtOAc extracts were evaporated in vacuo to give a residue (30.0 g). The residue was subjected to silica gel column chromatography (CC) with a gradient elution system of chloroform-methanol (100:0 → 0:100) to obtain ten fractions (A–J). Fraction E was subjected to preparative MPLC with a reversed-phased $C_{18}$ column (MeOH-$H_2O$, 0–60%) to obtain subfractions E01–E10. Fraction E06 was eluted with petroleum ether PE-EtOAc (3:1) and then subjected to Sephadex LH-20 CC ($CHCl_3$-MeOH, 1:1) to give **5** (5.0 mg). Fraction E10 eluted with PE-acetone (5:1). was further separated by preparative HPLC ($CH_3CN$-$H_2O$, 30%) to give **1** (1.5 mg) and **2** (6.0 mg). Fraction G was chromatographed over a silica gel column using PE-acetone (10:1 → 0:1) to produce fractions G01–G14. **6** (50.0 mg) and **7** (50.0 mg) were afforded from fraction G08 by preparative HPLC ($CH_3CN$-$H_2O$, 20%), compound **8** (2.0 mg) was also obtained by preparative HPLC ($CH_3CN$-$H_2O$, 30%) from the G03 fraction. Fraction G12 was subjected to a RP-18 column (MeOH-$H_2O$, 40%), then purified on a silica gel column (PE-acetone, 2:1) to afford **3** (20.0 mg). Fraction G13 was separated by repeated silica gel column chromatography (PE-acetone, 6:1 → 0:1) to yield fractions G131–G135. Fraction G131 was chromatographed on a RP-18 column (MeOH-$H_2O$, 50%) and then purified on a silica gel column (PE-EtOAc, 2:1) to yield **9** (60.0 mg) and **10** (5.0 mg). Compound **4** (15.0 mg) was obtained from fraction G132 after preparative HPLC ($CH_3CN$-$H_2O$, 10%), followed by Sephadex LH-20 CC ($CHCl_3$-MeOH, 1:1).

**Craterellone A (1):** white powder; $[\alpha]^{15}_{D}$ – 29.3 (*c* 0.20, MeOH); UV (MeOH) $\lambda_{max}$ (log ε) 268 (3.6), 238 (3.6), 206 (3.2) nm; IR (KBr) $\nu_{max}$ 3431, 1635 $cm^{-1}$; $^1H$ NMR (400 MHz) and $^{13}C$ NMR (100 MHz) data, see Tables 1 and 2, respectively; ESIMS (positive) *m/z* 283 $[M + Na]^+$; HRESIMS (positive) *m/z* 283.1313 $[M + Na]^+$ (calcd for $C_{16}H_{20}O_3Na$, 283.1310).

**Craterellone B (2):** yellow oil; $[\alpha]^{15}_{D}$ + 56.5 (*c* 0.33, MeOH); UV (MeOH) $\lambda_{max}$ (log ε) 234 (3.0), 201 (2.8) nm; IR (KBr) $\nu_{max}$ 3425, 2925, 1703 $cm^{-1}$; $^1H$ NMR (400 MHz) and $^{13}C$ NMR (100 MHz) data, see Tables 1 and 2, respectively; ESIMS (positive) *m/z* 259 $[M + Na]^+$; HRESIMS (positive) *m/z* 259.1678 $[M + Na]^+$ (calcd for $C_{15}H_{24}O_2Na$, 259.1673).

**Craterellone C (3):** yellow oil; $[\alpha]^{15}_{D}$ + 51.4 (*c* 0.40, MeOH); UV (MeOH) $\lambda_{max}$ (log ε) 203 (2.6) nm; IR (KBr) $\nu_{max}$ 3425, 1635 $cm^{-1}$; $^1H$ NMR (400 MHz) and $^{13}C$ NMR (100 MHz) data, see Tables 1 and 2, respectively; ESIMS (positive) *m/z* 321 $[M + Na]^+$; HRESIMS (positive) *m/z* 321.1668 $[M + Na]^+$ (calcd for $C_{16}H_{26}O_5Na$, 321.1677).

**Craterellone D (4):** yellow powder; $[\alpha]^{15}_{D}$ – 24.6 (*c* 0.40, MeOH); UV (MeOH) $\lambda_{max}$ (log ε) 254 (1.9), 201 (2.5) nm; IR (KBr) $\nu_{max}$ 3420, 2920, 1693, 1037 $cm^{-1}$; $^1H$ NMR (400 MHz) and $^{13}C$ NMR (100 MHz) data, see Tables 1 and 2, respectively; ESIMS (positive) *m/z* 305 $[M + Na]^+$; HRESIMS (positive) *m/z* 305.1727 $[M + Na]^+$ (calcd for $C_{16}H_{26}O_4Na$, 305.1728).

**Craterellone E (5):** yellow oil; $[\alpha]_D^{15}$ – 55.4 (*c* 0.27, MeOH); UV (MeOH) $\lambda_{max}$ (log ε) 232 (3.4), 194 (3.0) nm; IR (KBr) $\nu_{max}$ 3431, 1701, 1630 $cm^{-1}$; $^1H$ NMR (400 MHz) and $^{13}C$ NMR (100 MHz) data, see Tables 1 and 2, respectively; ESIMS (positive) *m/z* 287 $[M + Na]^+$; HRESIMS (positive) *m/z* 287.1621 $[M + Na]^+$ (calcd for $C_{16}H_{20}O_3Na$, 287.1623).

**Inhibitory Activities Against 11β-HSD1 Assay.** The inhibitory activities of the compounds on human or mouse 11β-HSD1 were determined using scintillation proximity assay (SPA). Microsomes containing 11β-HSD1 were used according to our previous studies.[5] The full-length cDNAs of human or murine 11β-HSD1 were isolated from the cDNA libraries provided by NIH Mammalian Gene Collection. The cDNAs were cloned into pcDNA3 expression vectors. HEK-293 cells were transfected with the pcDNA3-derived expression plasmid and selected by cultivation in the presence of 700 μg/mL of G418. The microsomal fraction overexpressing 11β-HSD1 was prepared from the HEK-293 cells, which were stable transfected with 11β-HSD1. The fraction was then used as the enzyme source for SPA. Microsomes containing human or mouse 11β-HSD1 were incubated with NADPH and $[^3H]$cortisone. The product, $[^3H]$cortisol, was specifically captured by a monoclonal antibody coupled to protein A-coated SPA beads. All tests were done in twice with glycyrrhizinic acid as a positive control. $IC_{50}$ (X ± SD, n = 2) values were calculated by using Prism Version 4 (GraphPad Software, San Diego, CA).

**Cytotoxic Assay.** All compounds were evaluated for their cytotoxicity against five human cancer cell lines, breast cancer SK-BR-3, hepatocellular carcinoma SMMC-7721, human myeloid leukemia HL-60, pancreatic cancer PANC-1, and lung cancer A-549 cells. Cells were cultured in RPMI-1640 or in DMEM medium (Hyclone, USA), supplemented with 10% fetal bovine serum (Hyclone, USA) in 5% $CO_2$ at 37 °C. The cytotoxicity assay was performed according to 3-(4,5-dimethylthiazol-2-yl)-2,5-diphenyl tetrazolium bromide (MTT) method in 96-well microplates.[6] Briefly, 100 μL of adherent cells were seeded into each well of 96-well cell culture plates and allowed to adhere for 12 h before addition of test compounds, while suspended cells were seeded just before drug addition with initial density of $1 \times 10^5$ cells/mL. Each tumor cell line was exposed to the test compound at concentrations of 0.0625, 0.32, 1.6, 8, and 40 μM in triplicates for 48 h, and all tests were done in twice with cisplatin (Sigma, USA) as a positive control. After compound treatment, cell viability was detected and a cell growth curve was graphed. $IC_{50}$ values were calculated by Reed and Muench's method.[7]

## Electronic Supplementary Material

Supplementary material is available in the online version of this article at http://dx.doi.org/ 10.1007/s13659-012-0057-5 and is accessible for authorized users.

## Acknowledgments

This work was financially supported by National Basic Research Program of China (973 Program, 2009CB522300), the National Natural Science Foundation of China (30830113, U1132607).

**Open Access** This article is distributed under the terms of the Creative Commons Attribution License which permits any use, distribution, and reproduction in any medium, provided the original author(s) and source are credited.

## Electronic supplementary material

Supplementary material, approximately 40 KB.
