# Supplementary material for: Five new polyketides from the basidiomycete Craterellus odoratus
Source: Nat Prod Bioprospect. 2012 Aug 7;2(4):170–3. doi: 10.1007/s13659-012-0057-5 (PMC4131629; doi:10.1007/s13659-012-0057-5)
Supplement: Supplementary file 1 — Supplementary material, approximately 40 KB. [file 13659_2012_57_MOESM1_ESM.pdf]

Electronic Supplementary Material

Five new polyketides from the basidiomycete *Craterellus odoratus*

Hua GUO,[a,b,c] Tao FENG,[a] Zheng-Hui LI,[a] and Ji-Kai LIU[a,]*

[a]State Key Laboratory of Phytochemistry and Plant Resources in West China, Kunming Institute of Botany, Chinese Academy of Sciences, Kunming 650201, China

[b]School of Chemistry and Life Science, Anshan Normal College, Anshan 114005, China

[c]Graduate University of Chinese Academy of Sciences, Beijing 100049, China

Received 9 July 2012; Accepted 30 July 2012

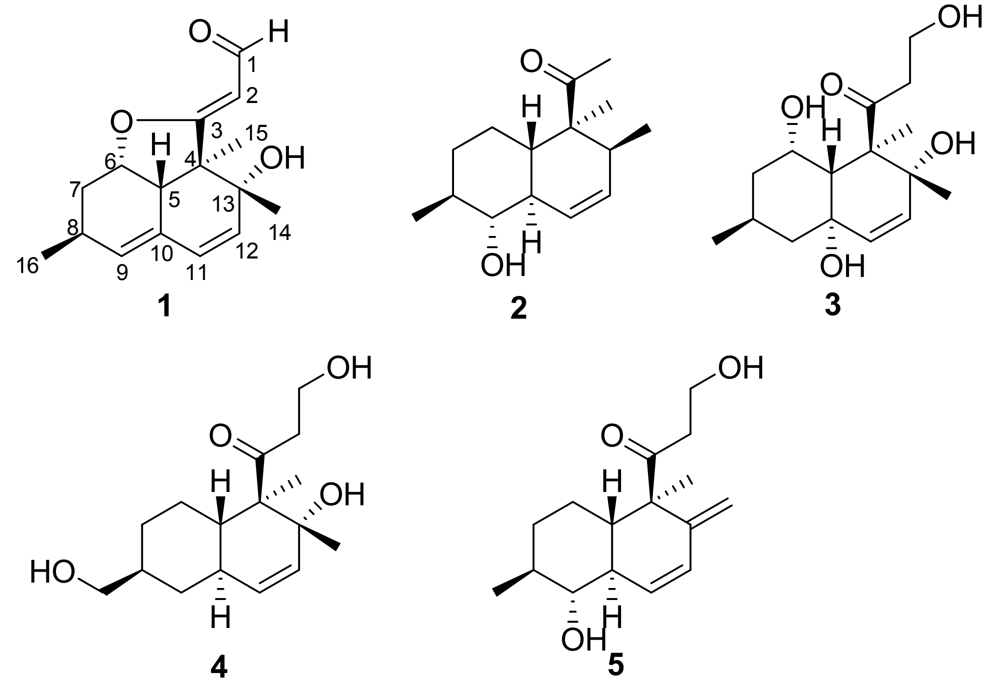

Structures of compounds **1**–**5**

*To whom correspondence should be addressed. E-mail: jkliu@mail.kib.ac.cn

Contents

Figure 1S–7S. NMR and MS of craterellone A (**1**).

Figure 8S–14S. NMR and MS of craterellone B (**2**).

Figure 15S–21S. NMR and MS of craterellone C (**3**).

Figure 22S–28S. NMR and MS of craterellone D (**4**).

Figure 29S–35S. NMR and MS of craterellone E (**5**).

Figure 1S. ^{1}H NMR of craterellone A (**1**).

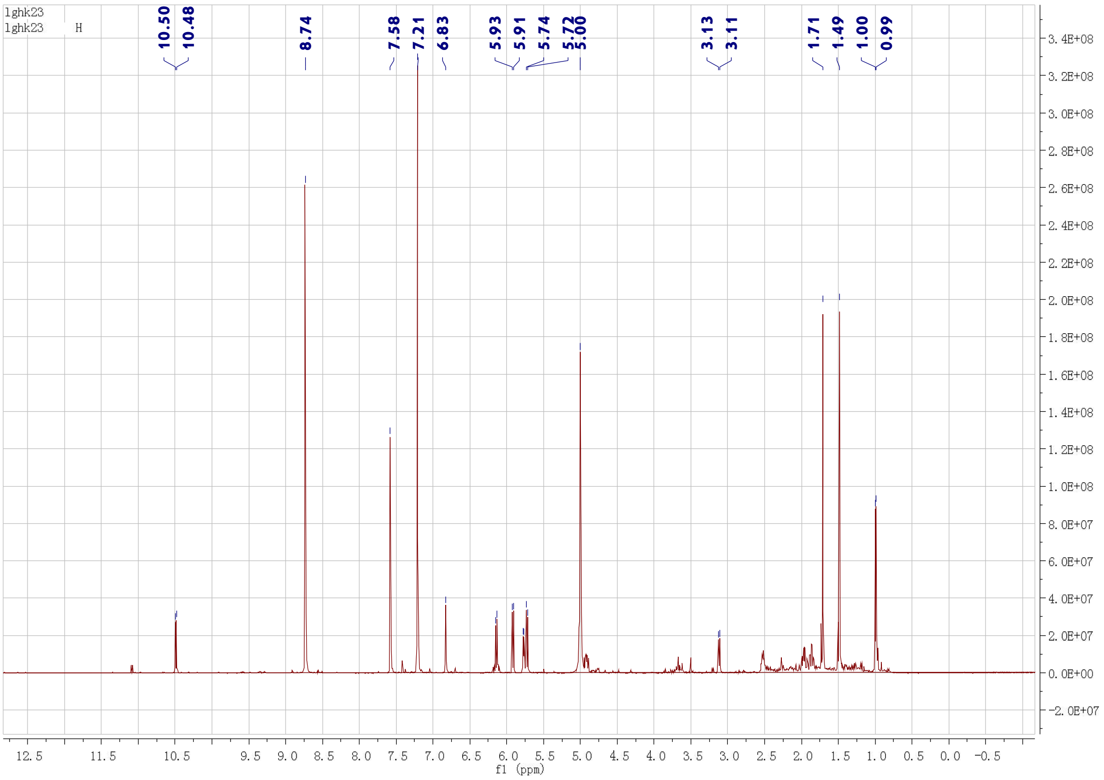

Figure 2S. ^{13}C NMR of craterellone A (**1**).

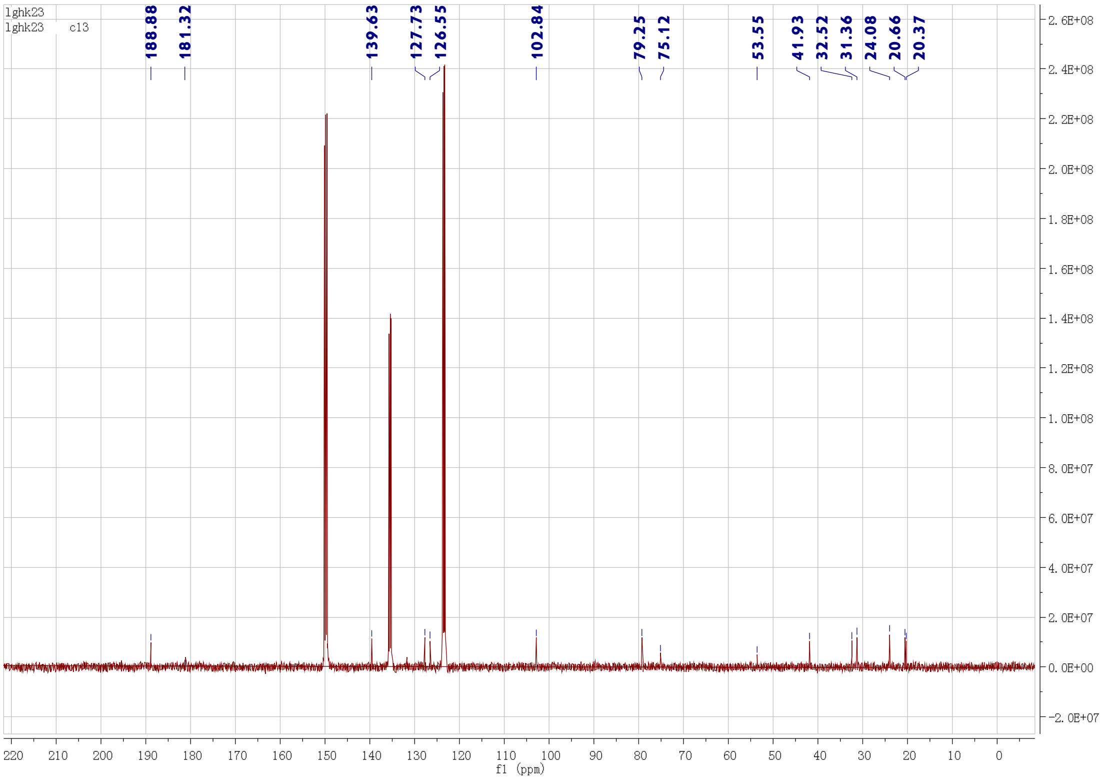

Figure 3S. HSQC of craterellone A (**1**).

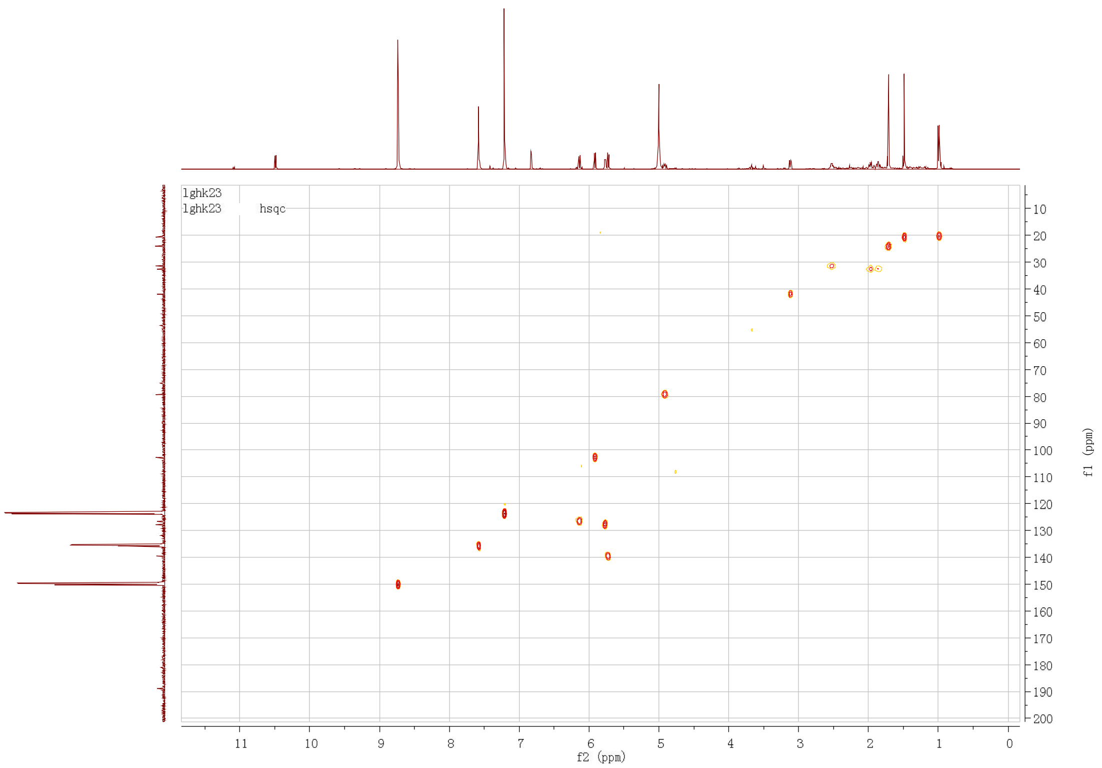

Figure 4S. HMBC craterellone A (**1**)

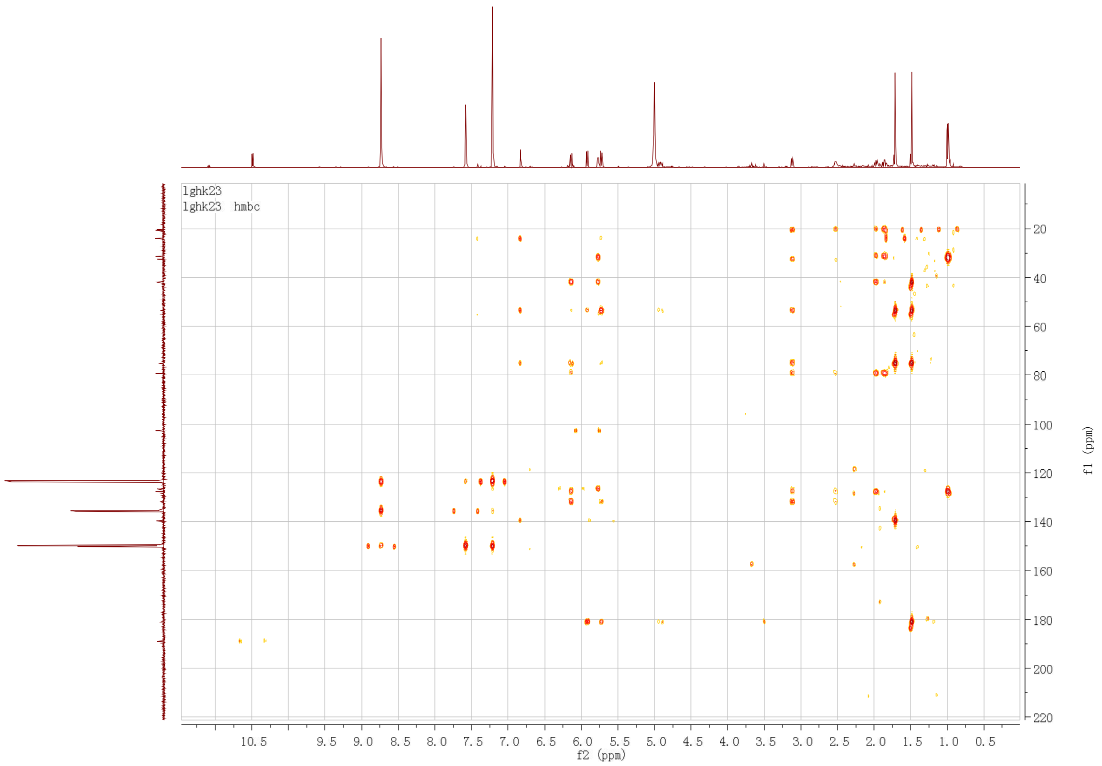

Figure 5S. ^{1}H-^{1}H COSY of craterellone A (**1**).

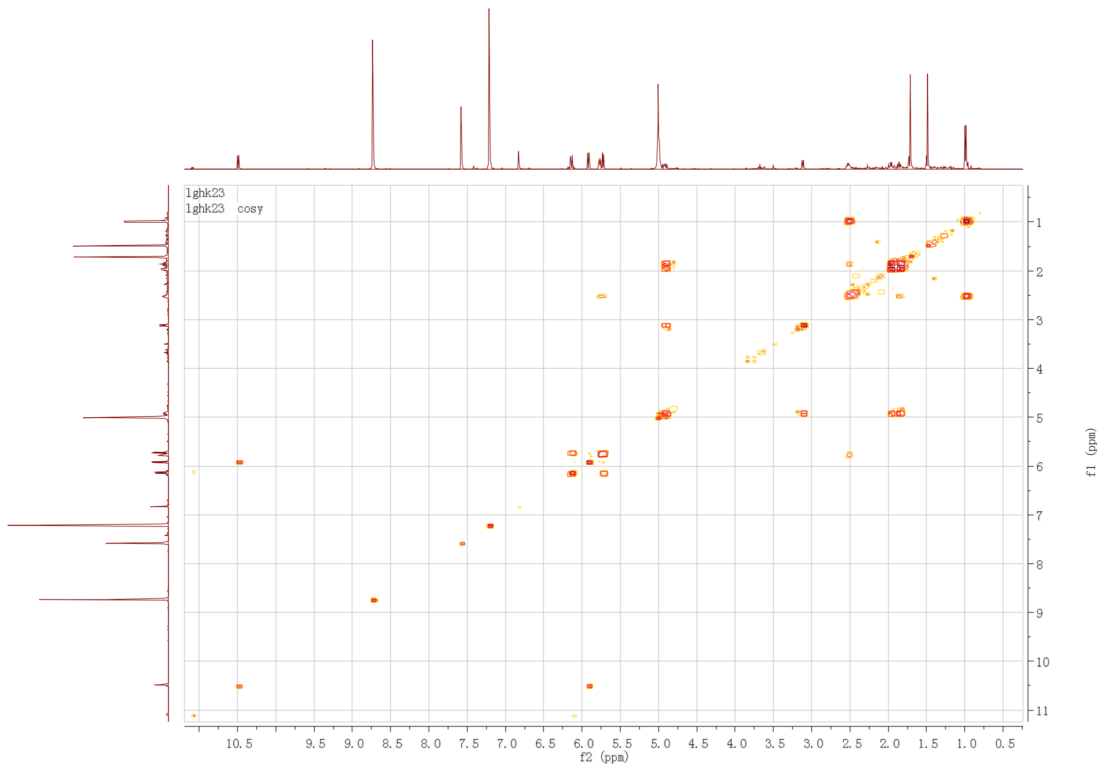

Figure 6S. ROESY of craterellone A (**1**).

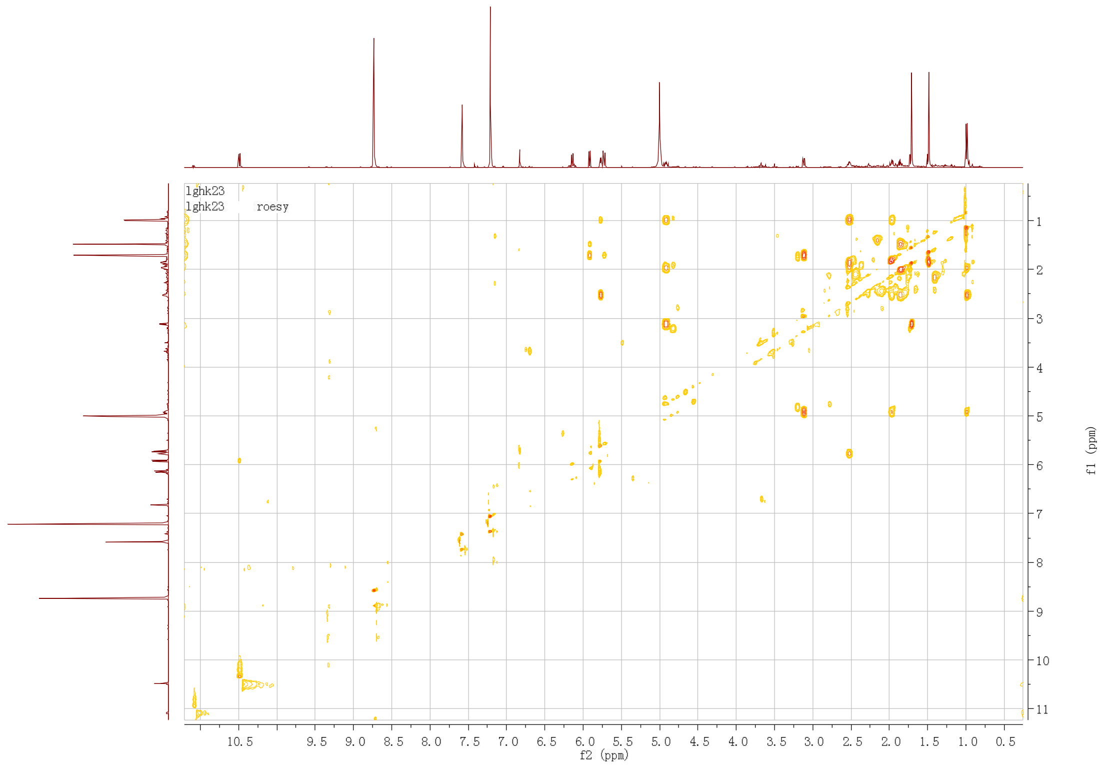

Figure 7S. HRESIMS of craterellone A (**1**).

Acq. Date: Wednesday, January 11, 2012   Acq. Time: 09:46

Sample Name: 120112ESIA   Lghk23

Elemental composition calculator

Target m/z: -283.1313 amu
Tolerance: +10.0000 ppm
Result type: Elemental
Max num of results: 1000
Min DBE: -10.0000   Max DBE: +60.0000
Electron state: OddAndEven
Num of charges: 0
Add water: N/A
Add proton: N/A
File Name: 120112ESIA   Lghk23.wiff

	Elements	Min Number	Max Number
1	2H	0	0
2	Br	0	0
3	C	0	200
4	Cl	0	0
5	F	0	0
6	H	0	400
7	I	0	0
8	K	0	0
9	N	0	0
10	Na	1	1
11	O	1	5

Figure 8S. ^{1}H NMR of craterellone B (**2**).

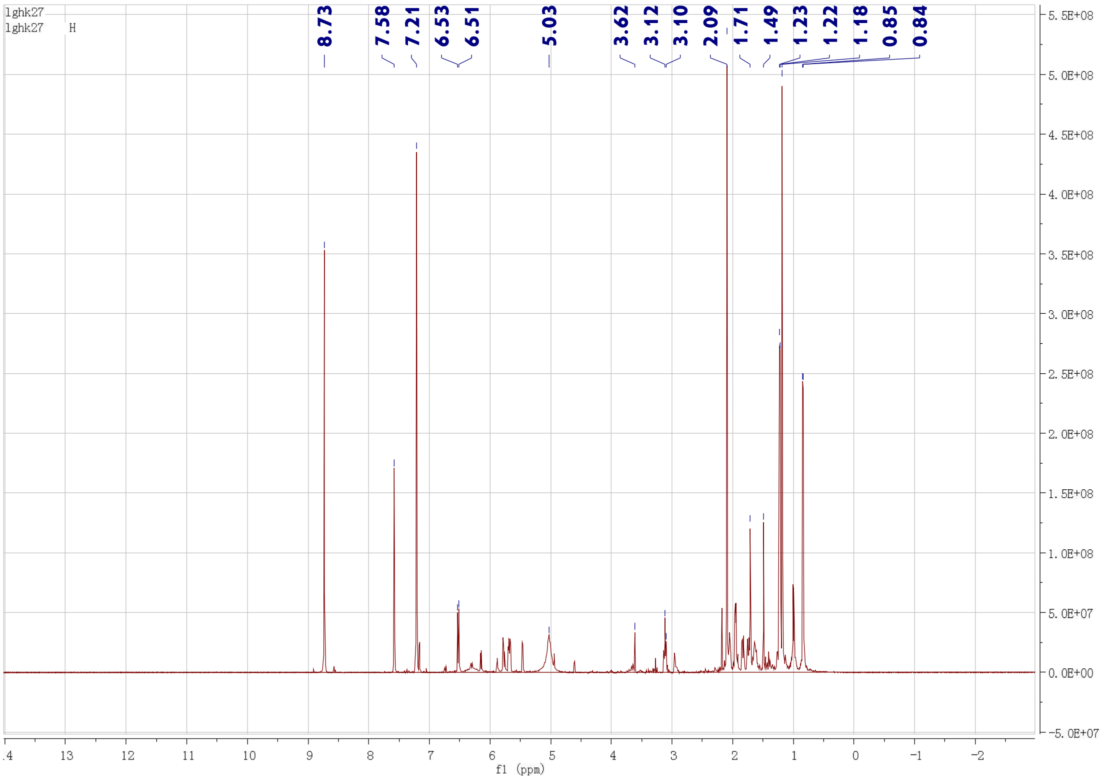

Figure 9S. ^{13}C NMR of craterellone B (**2**).

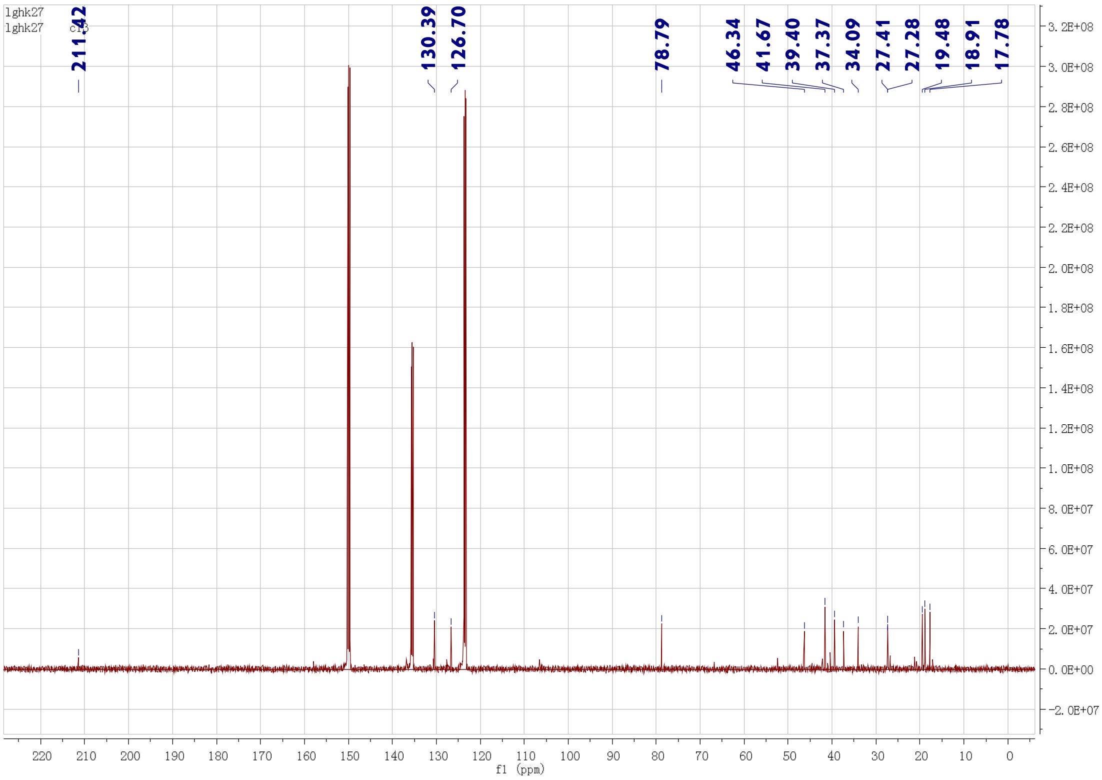

Figure 10S. HSQC of craterellone B (**2**).

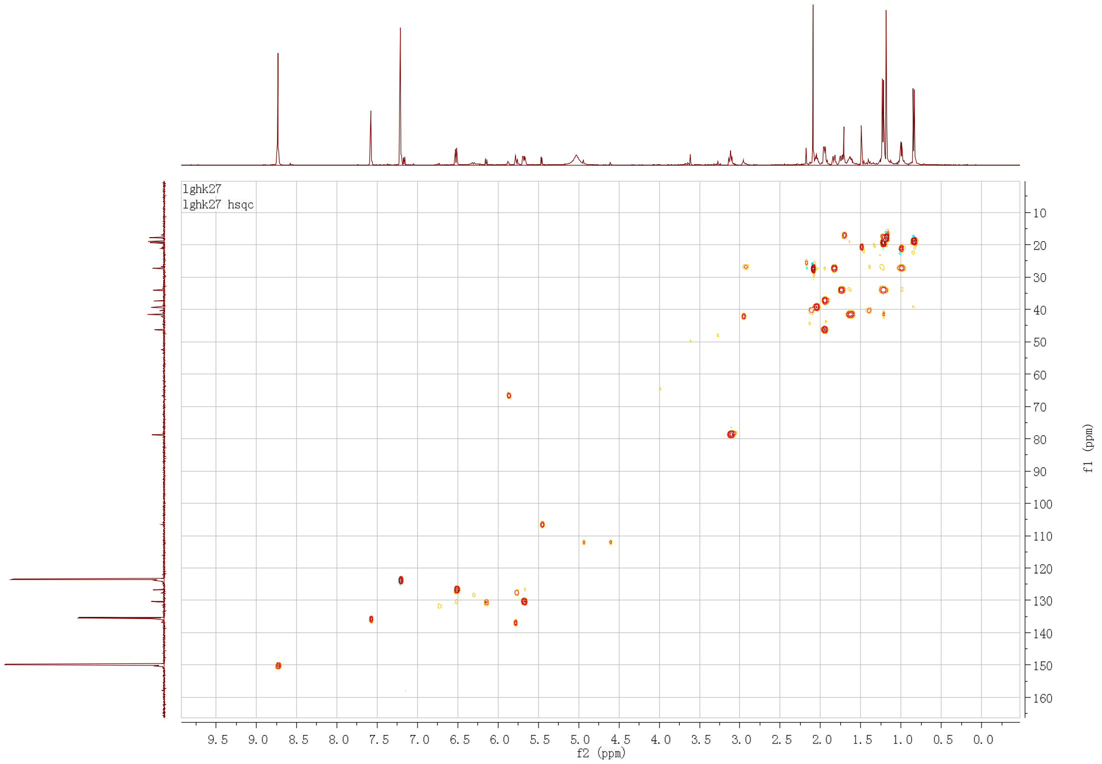

Figure 11S. HMBC of craterellone B (**2**).

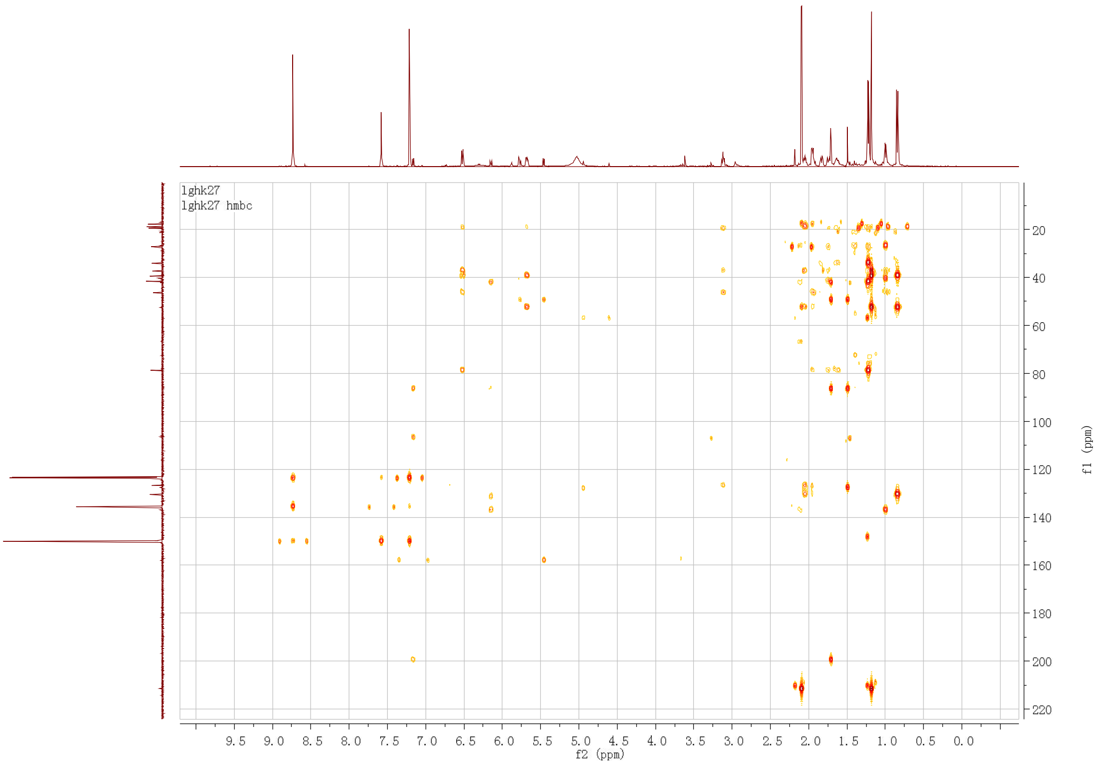

Figure 12S. ^{1}H-^{1}H COSY of craterellone B (**2**).

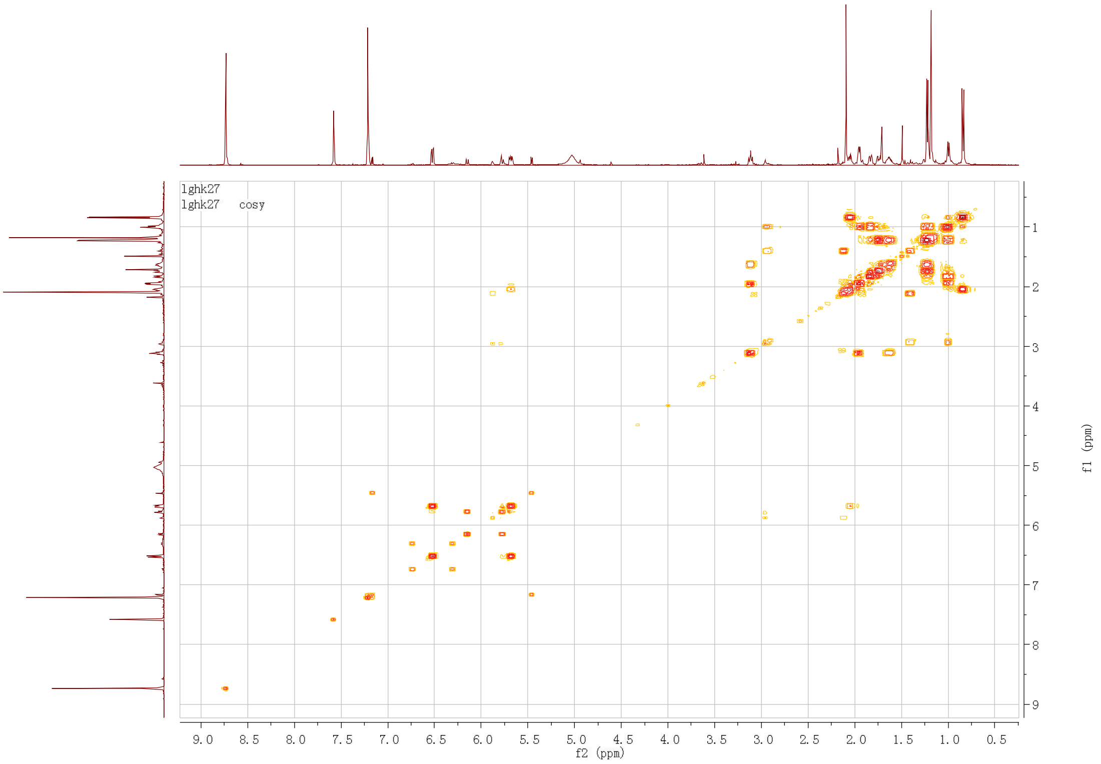

Figure 13S. ROESY of craterellone B (**2**).

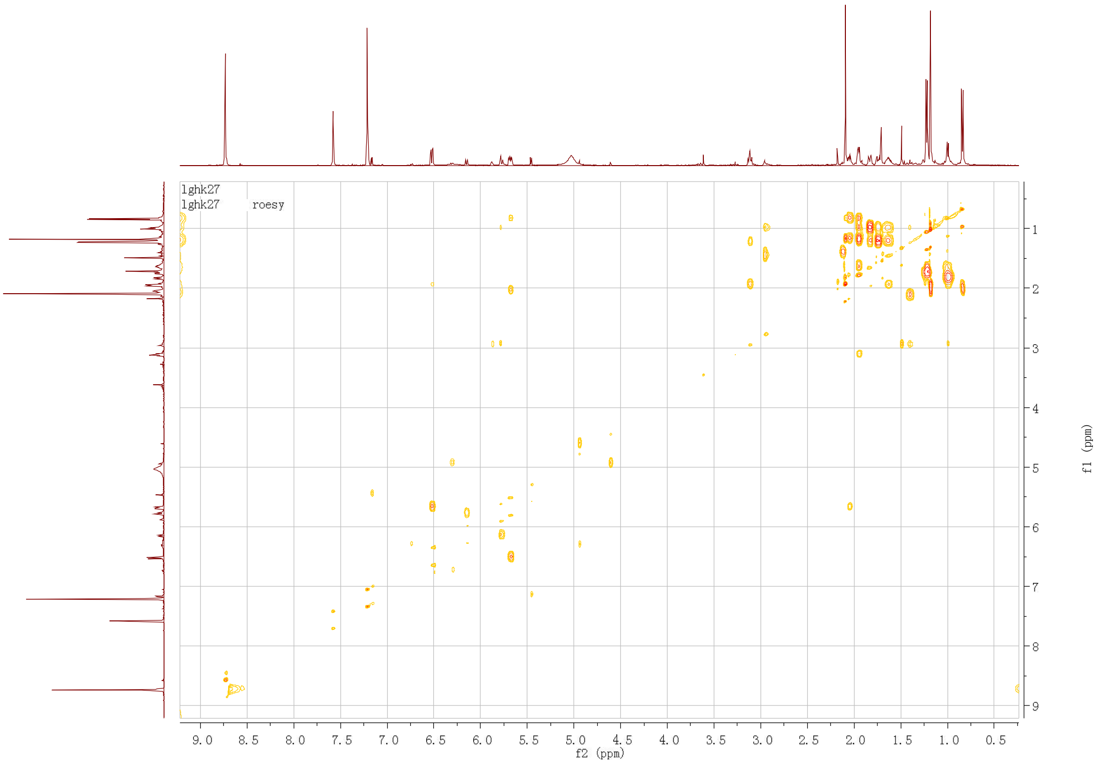

Figure 14S. HRESIMS of craterellone B (**2**).

Acq. Date: Wednesday, January 11, 2012   Acq. Time: 09:49

Sample Name: 120112ESIA lghk27

Elemental composition calculator

Target m/z: +259.1678 amu
Tolerance: +10.0000 ppm
Result type: Elemental
Max num of results: 1000
Min DBE: -10.0000 Max DBE: +60.0000
Electron state: OddAndEven
Num of charges: 0
Add water: N/A
Add proton: N/A
File Name: 120112ESIA lghk27.wiff

	Elements	Min Number	Max Number
1	2H	0	0
2	Br	0	0
3	C	0	200
4	Cl	0	0
5	F	0	0
6	H	0	400
7	I	0	0
8	K	0	0
9	N	0	0
10	Na	1	1
11	O	1	5

Figure 15S. ^{1}H NMR of craterellone C (**3**).

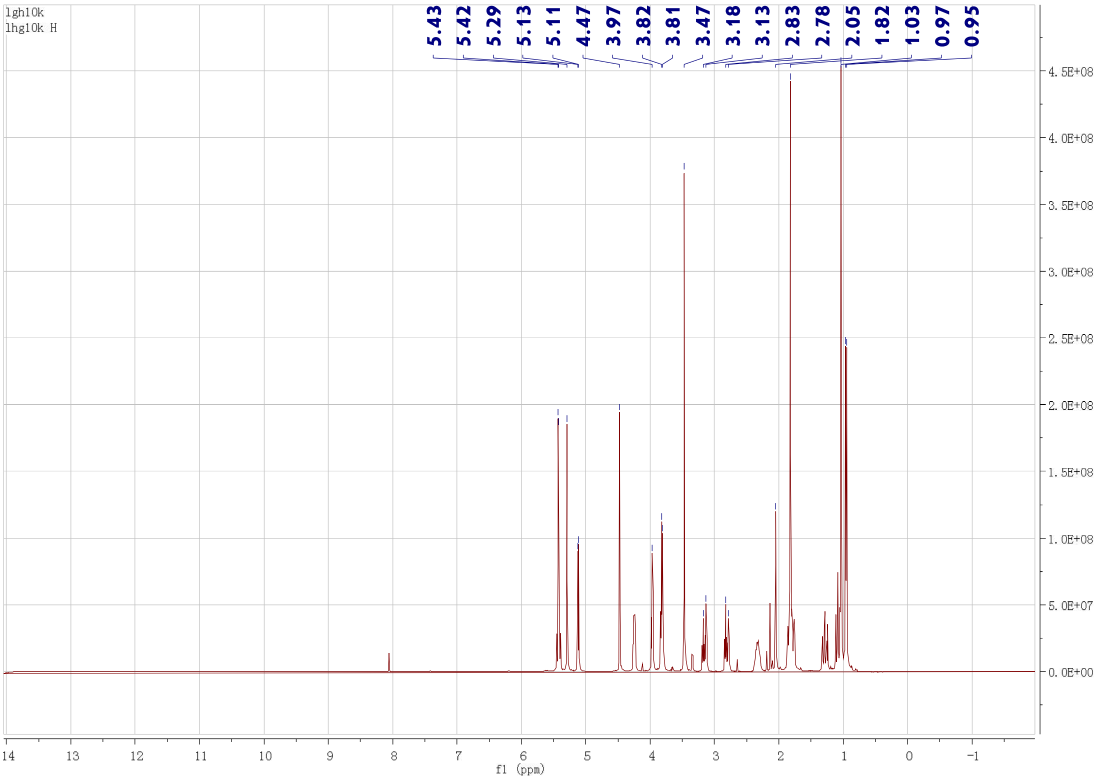

Figure 16S. ^{13}C NMR of craterellone C (**3**).

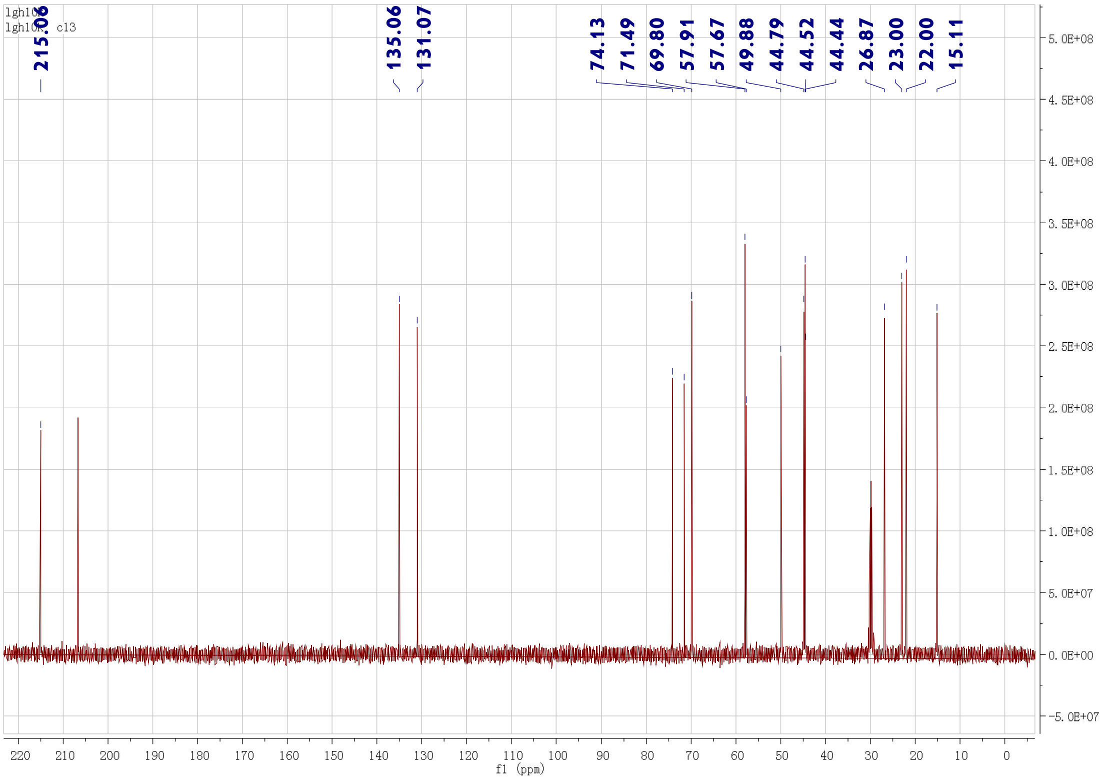

Figure 17S. HSQC of craterellone C (**3**).

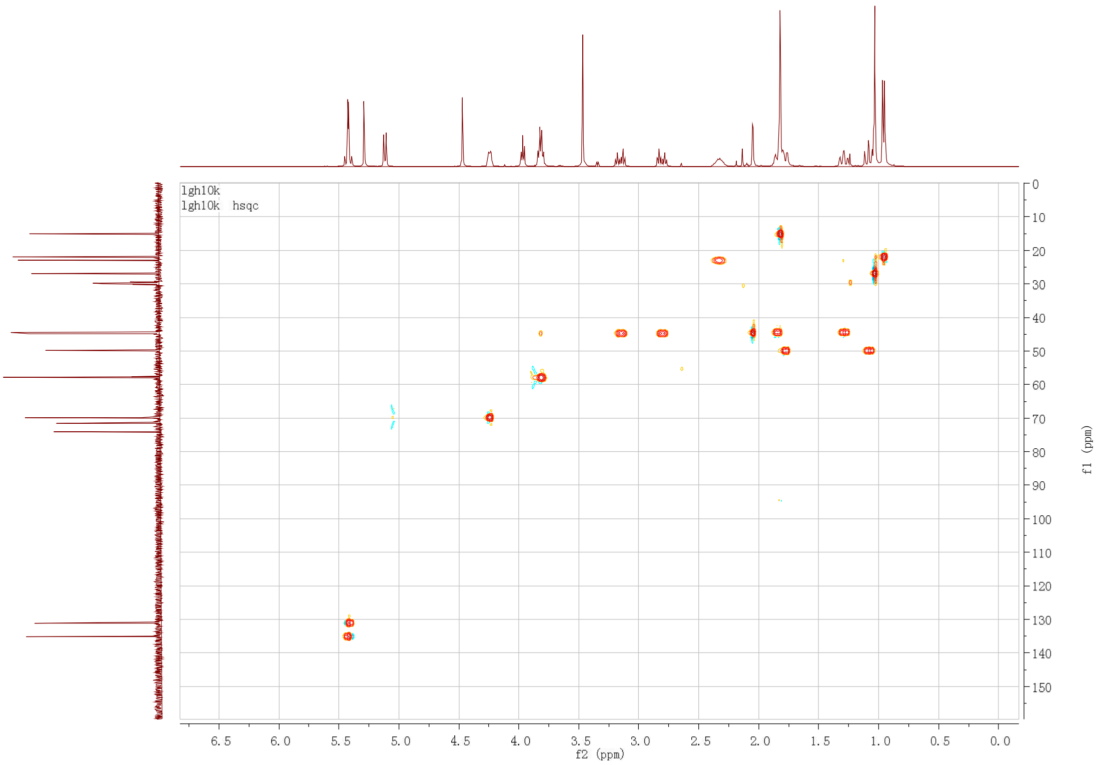

Figure 18S. HMBC of craterellone C (**3**).

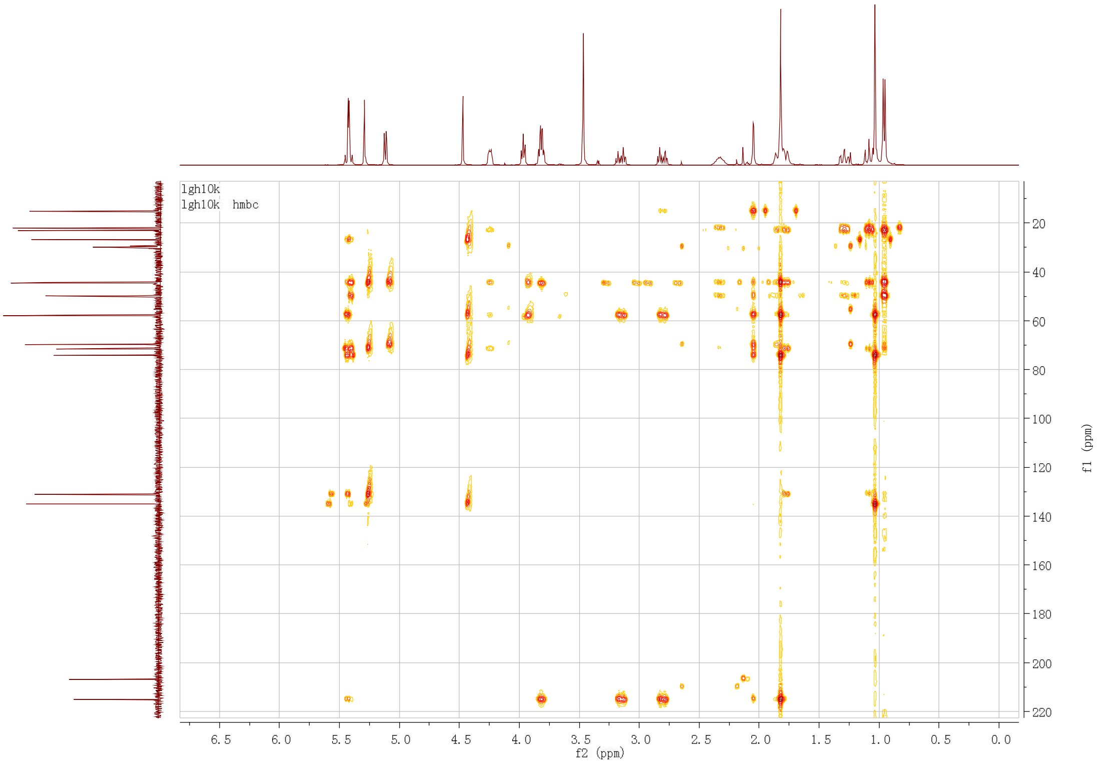

Figure 19S. ^{1}H-^{1}H COSY of craterellone C (**3**).

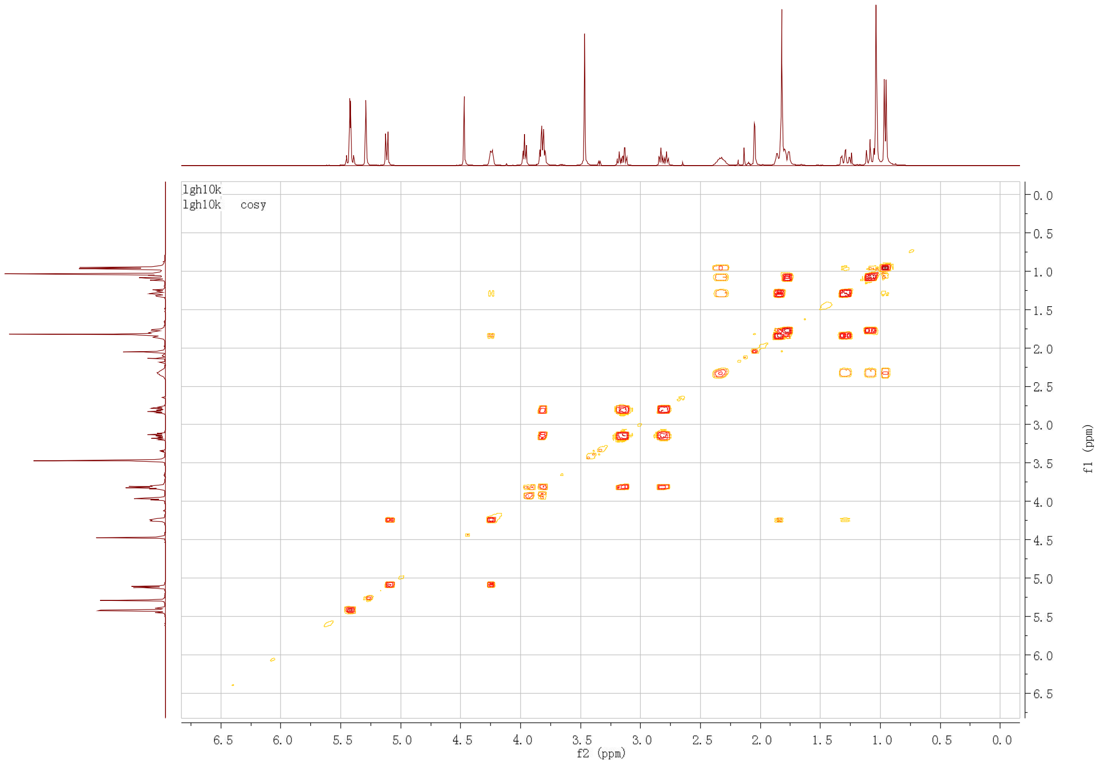

Figure 20S. ROESY of craterellone C (**3**).

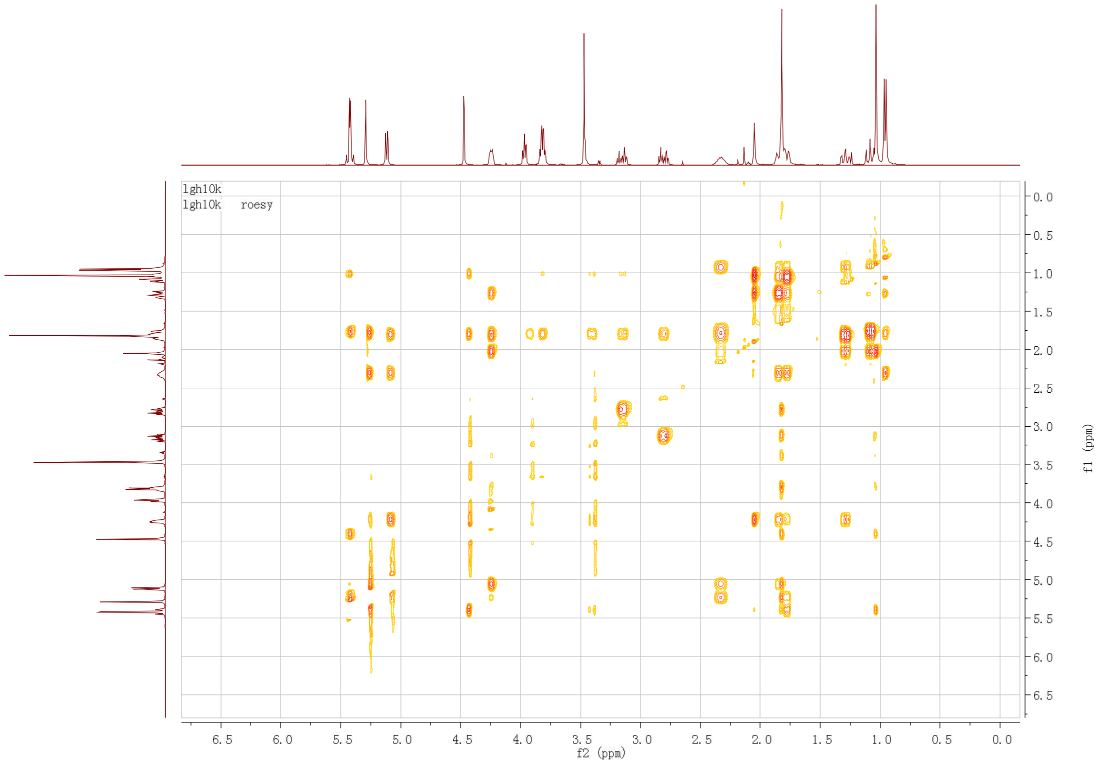

Figure 21S. HRESIMS of craterellone C (**3**).

Acq. Date: Wednesday, January 11, 2012 Acq. Time: 09:34

Sample Name: 120112ESIA Lgh10k

Elemental composition calculator

Target m/z: +321.1668 amu
Tolerance: ±10.0000 ppm
Result type: Elemental
Max num of results: 1000
Min DBE: -10.0000 Max DBE: +60.0000
Electron state: OddAndEven
Num of charges: 0
Add water: N/A
Add proton: N/A
File Name: 120112ESIA Lgh10k.wiff

	Elements	Min Number	Max Number
1	2H	0	0
2	Br	0	0
3	C	0	200
4	Cl	0	0
5	F	0	0
6	H	0	400
7	I	0	0
8	K	0	0
9	N	0	0
10	Na	1	1
11	O	1	5

Figure 22S. ^{1}H NMR of craterellone D (**4**).

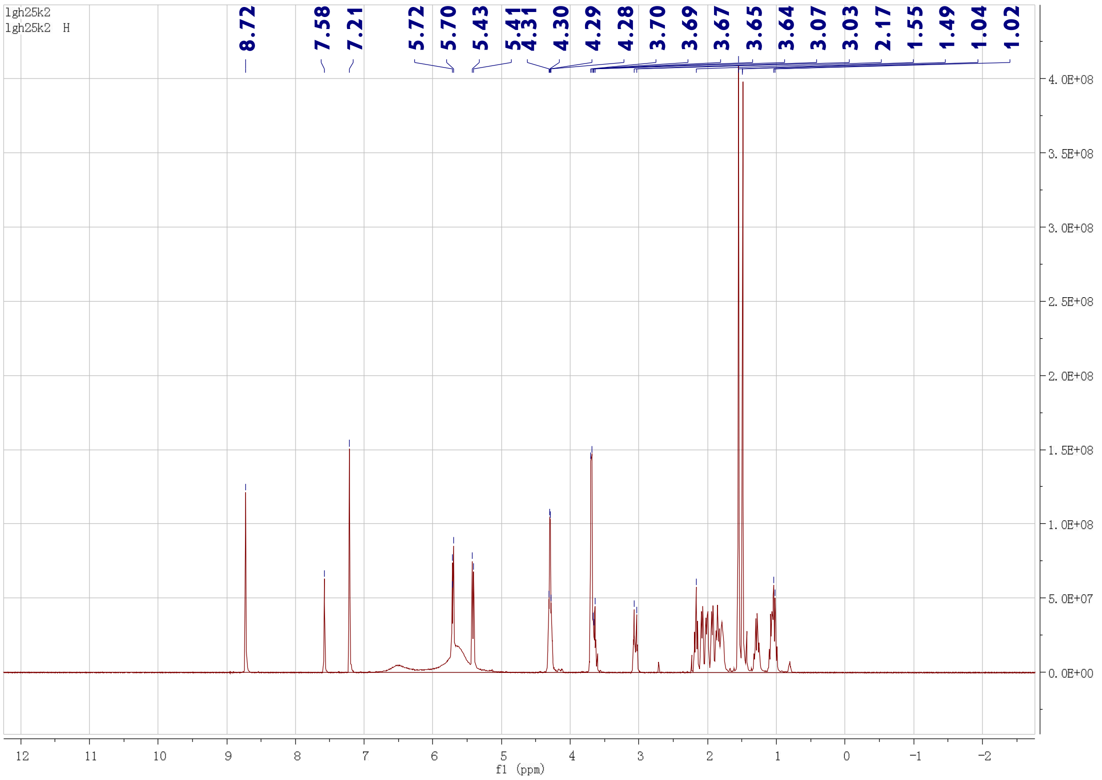

Figure 23S. ^{13}C NMR of craterellone D (**4**).

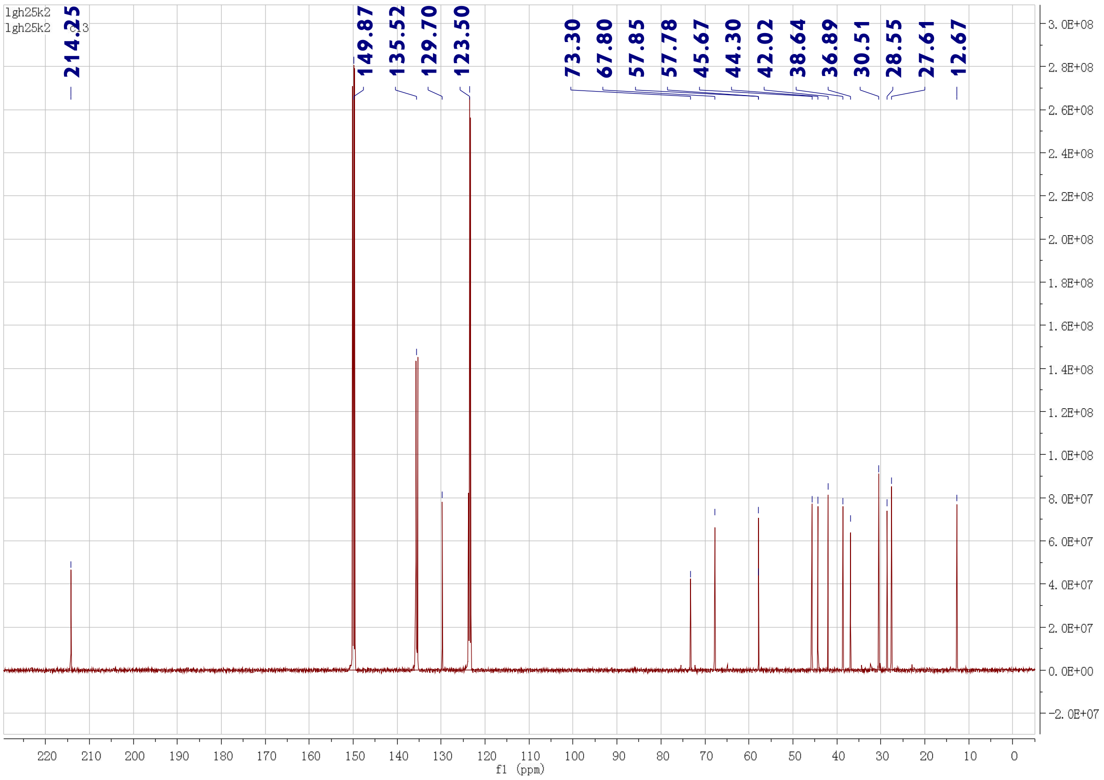

Figure 24S. HSQC of craterellone D (**4**).

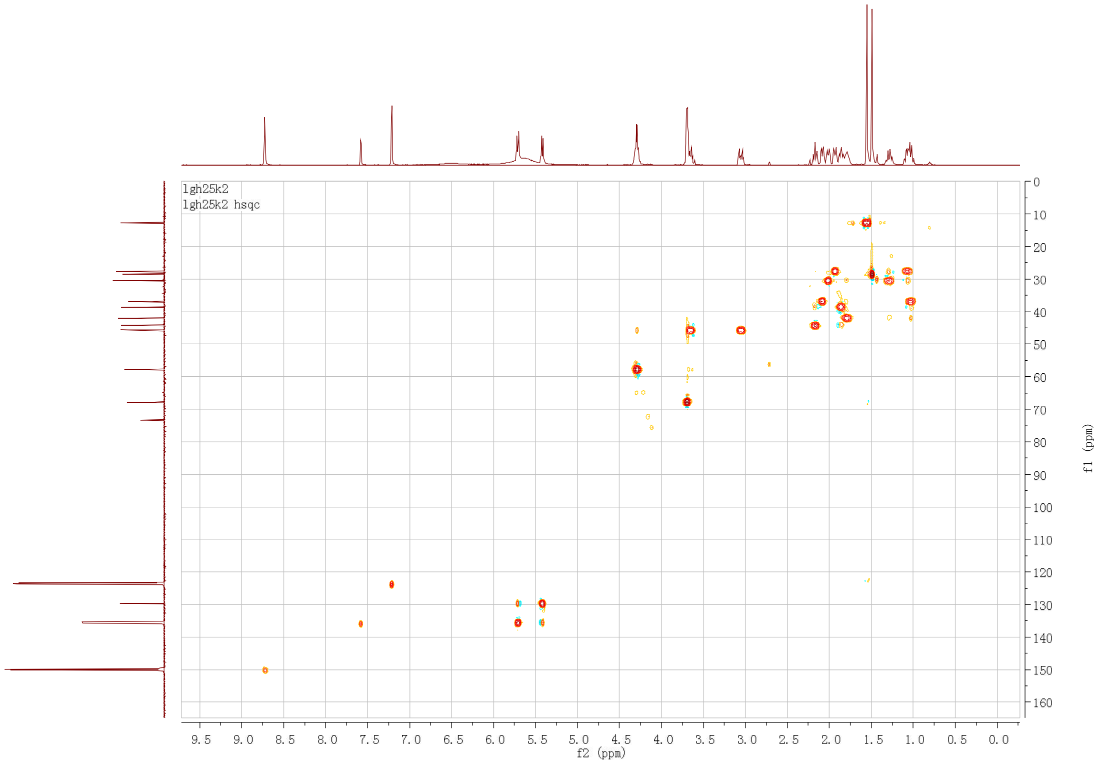

Figure 25S. HMBC of craterellone D (**4**).

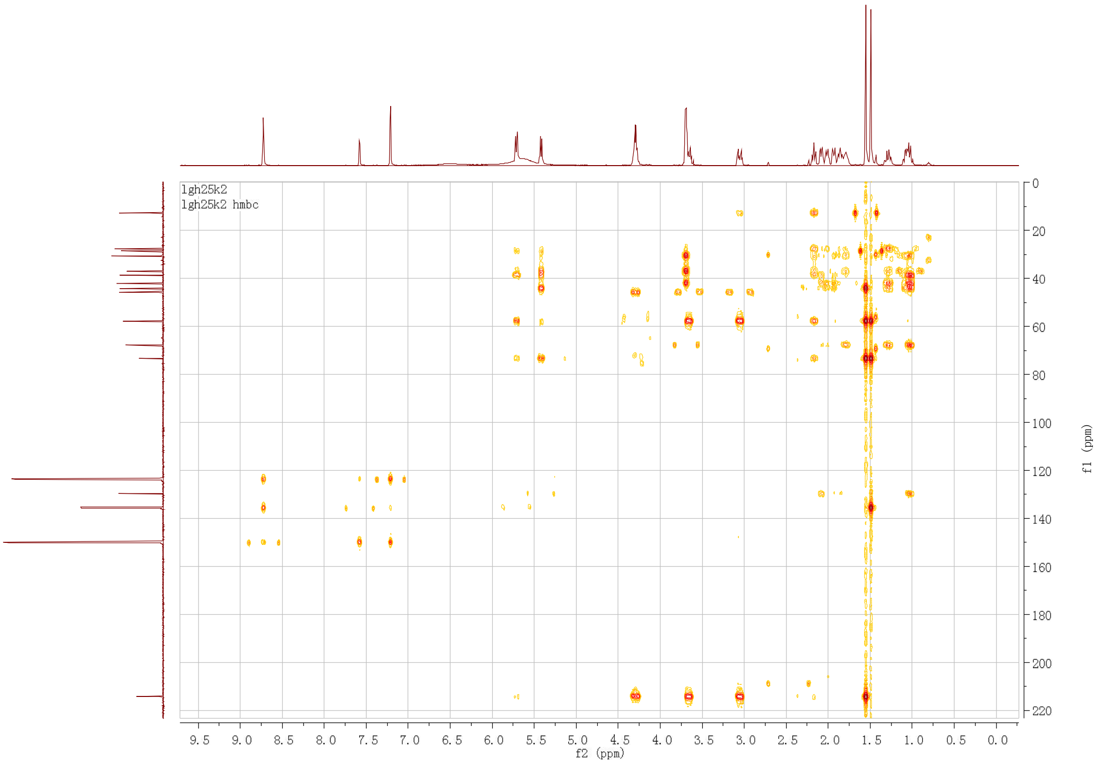

Figure 26S. ^{1}H-^{1}H COSY of craterellone D (**4**).

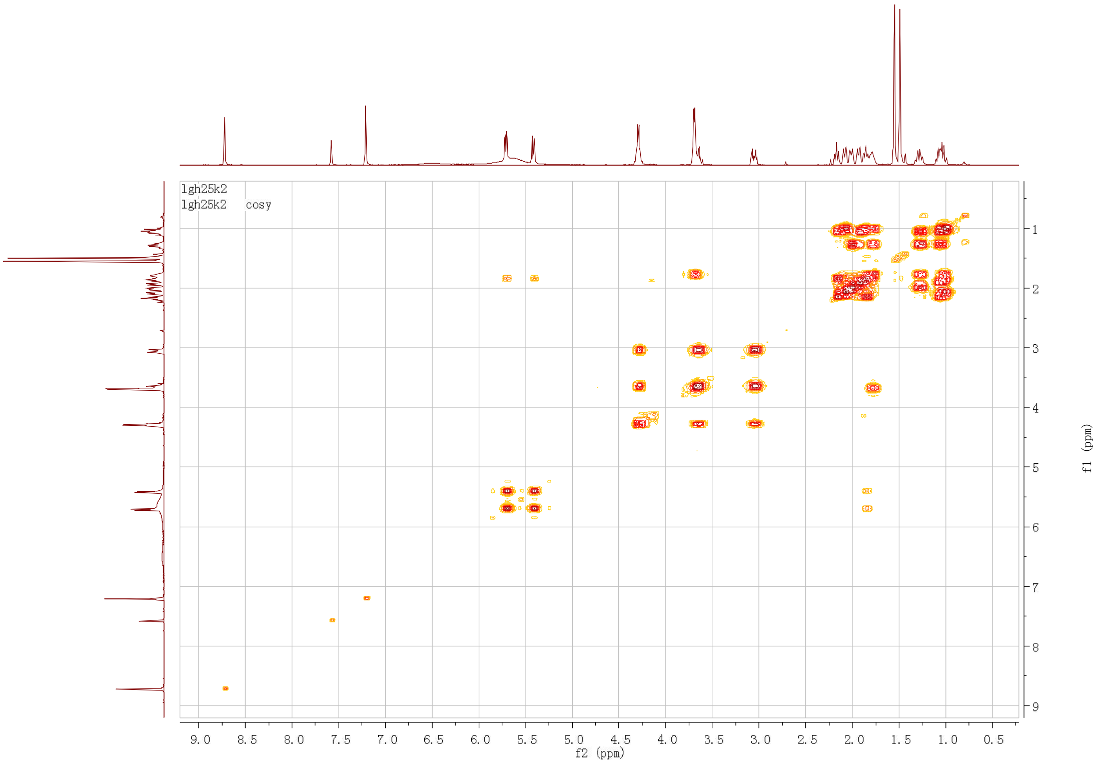

Figure 27S. ROESY of craterellone D (**4**).

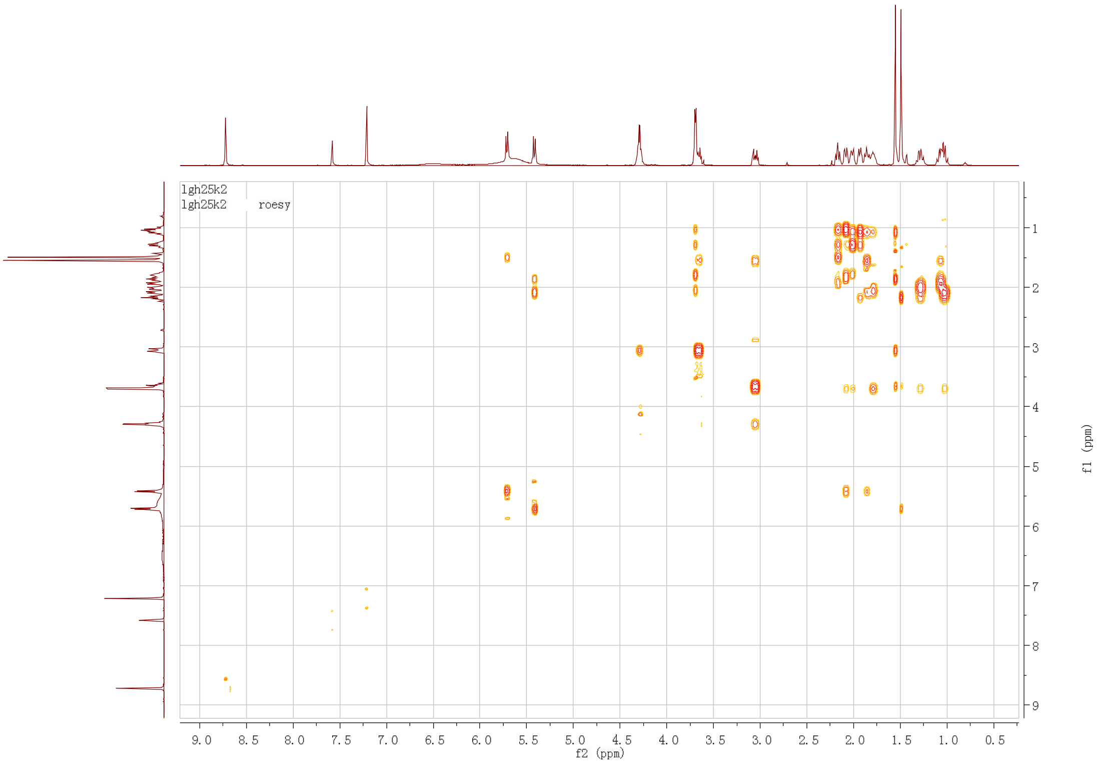

Figure 28S. HRESIMS of craterellone D (**4**).

Acq. Date: Wednesday, January 11, 2012　　Acq. Time: 09:42

Sample Name: 120112ESIA　Lgh25k2

Elemental composition calculator

Target m/z: +305.1727 amu
Tolerance: ±10.0000 ppm
Result type: Elemental
Max num of results: 1000
Min DBE: -10.0000　Max DBE: +60.0000
Electron state: OddAndEven
Num of charges: 0
Add water: N/A
Add proton: N/A
File Name: 120112ESIA　lgh25k2.wiff

	Elements	Min Number	Max Number
1	2H	0	0
2	Br	0	0
3	C	0	200
4	Cl	0	0
5	F	0	0
6	H	0	400
7	I	0	0
8	K	0	0
9	N	0	0
10	Na	1	1
11	O	1	5

Figure 29S. ^{1}H NMR of craterellone E (**5**).

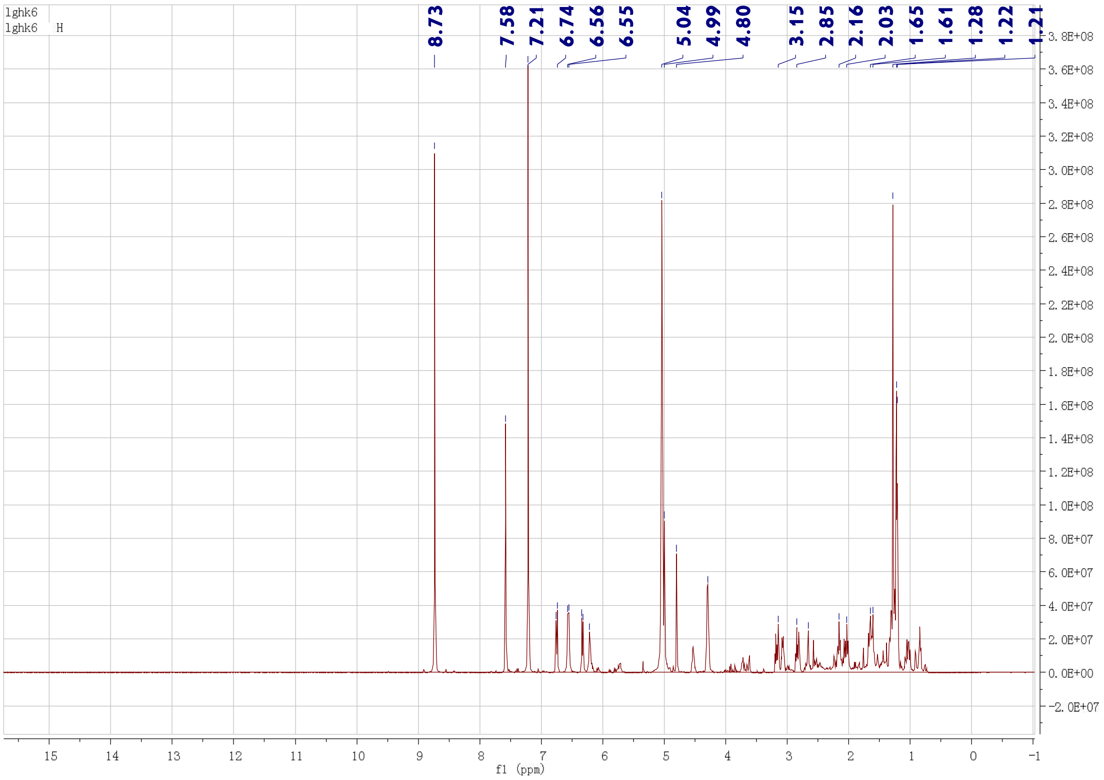

Figure 30S. ^{13}C NMR of craterellone E (**5**).

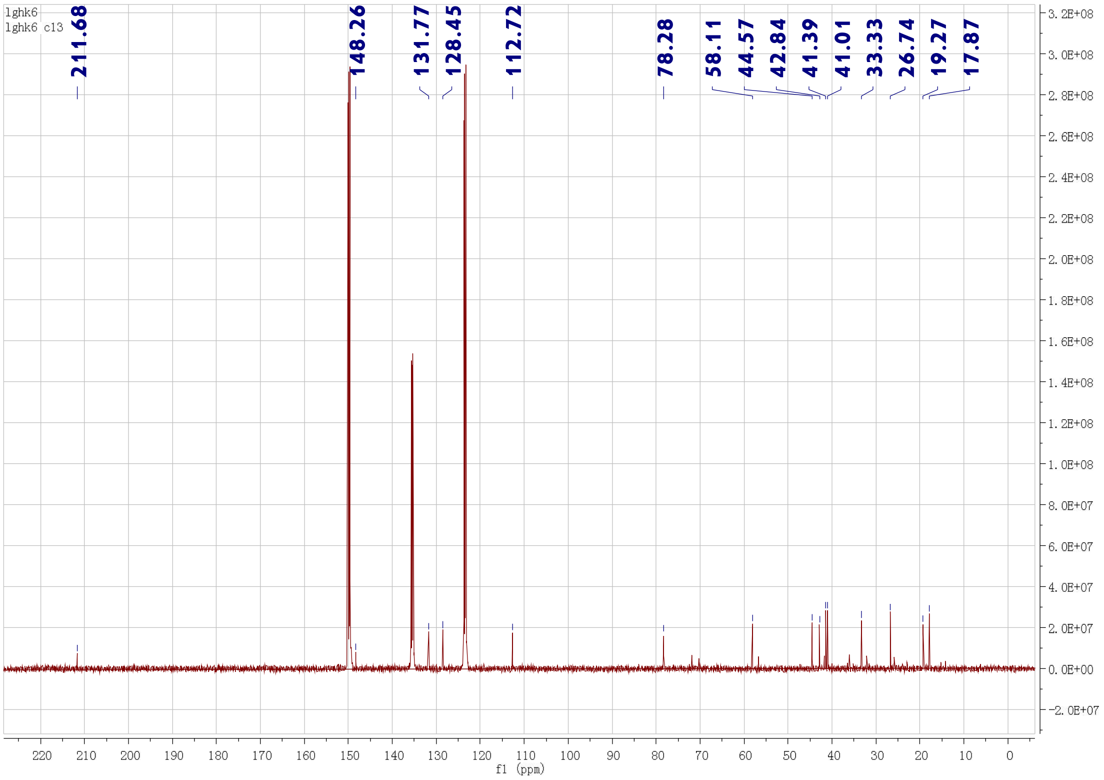

Figure 31S. HSQC of craterellone E (**5**).

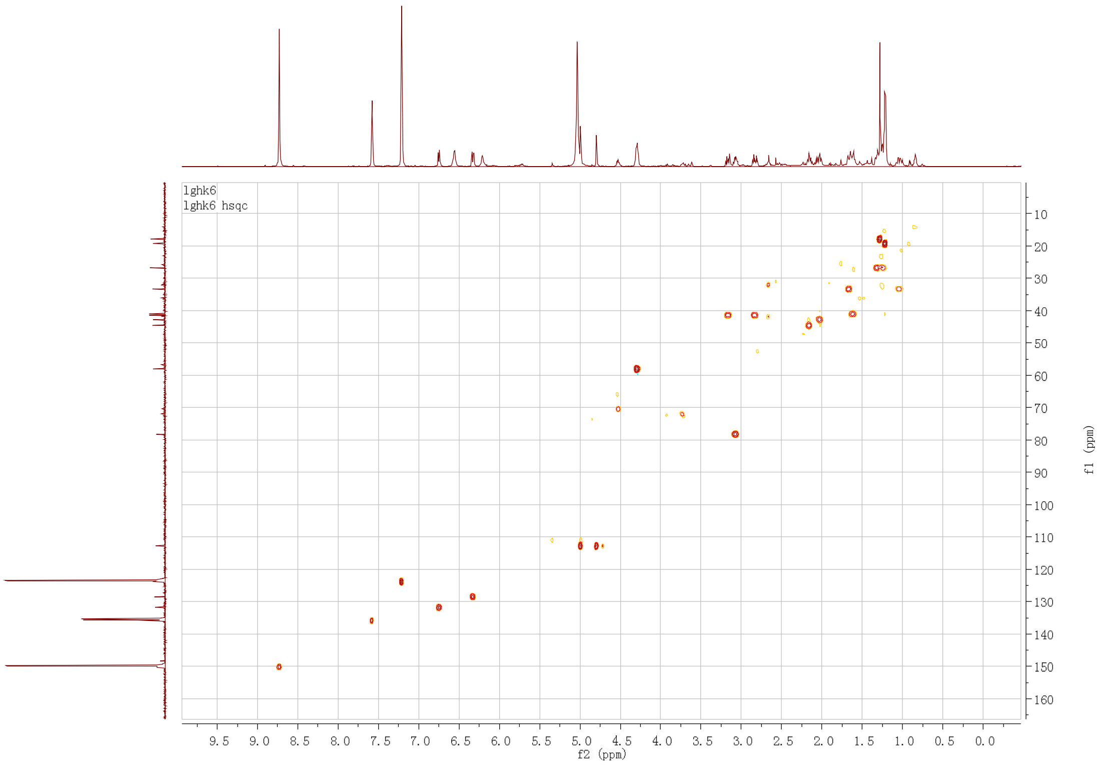

Figure 32S. HMBC of craterellone E (**5**).

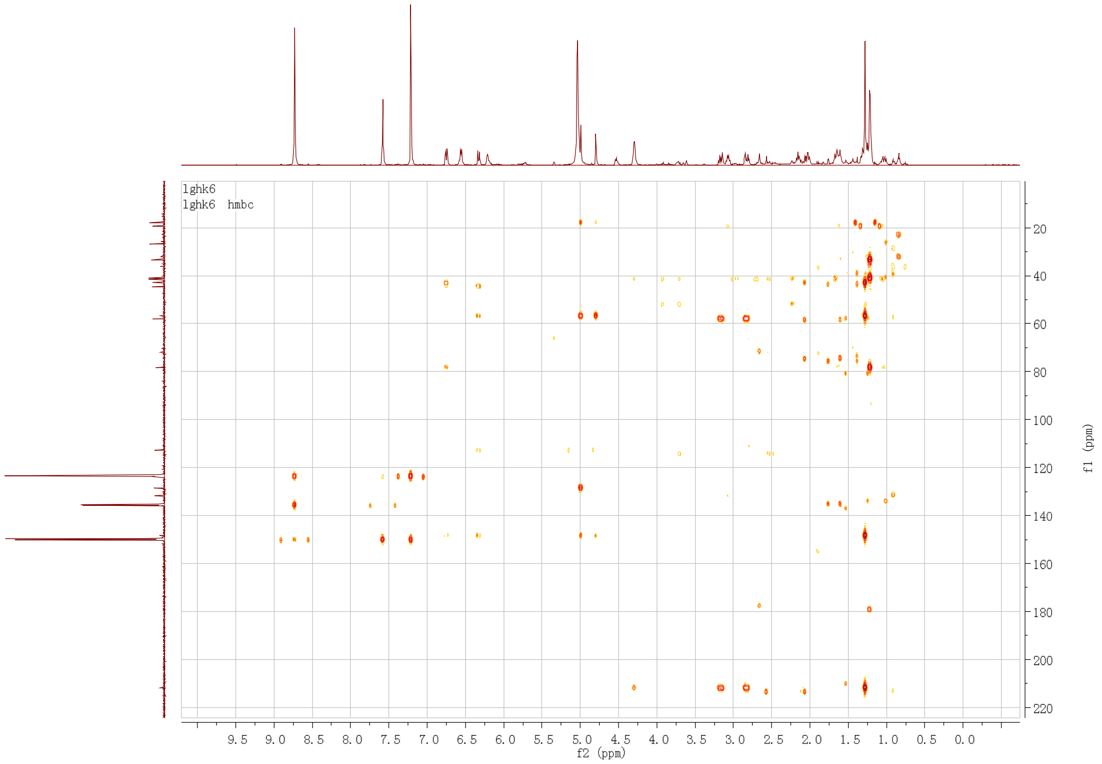

Figure 33S. ^1H-^1H COSY of craterellone E (**5**).

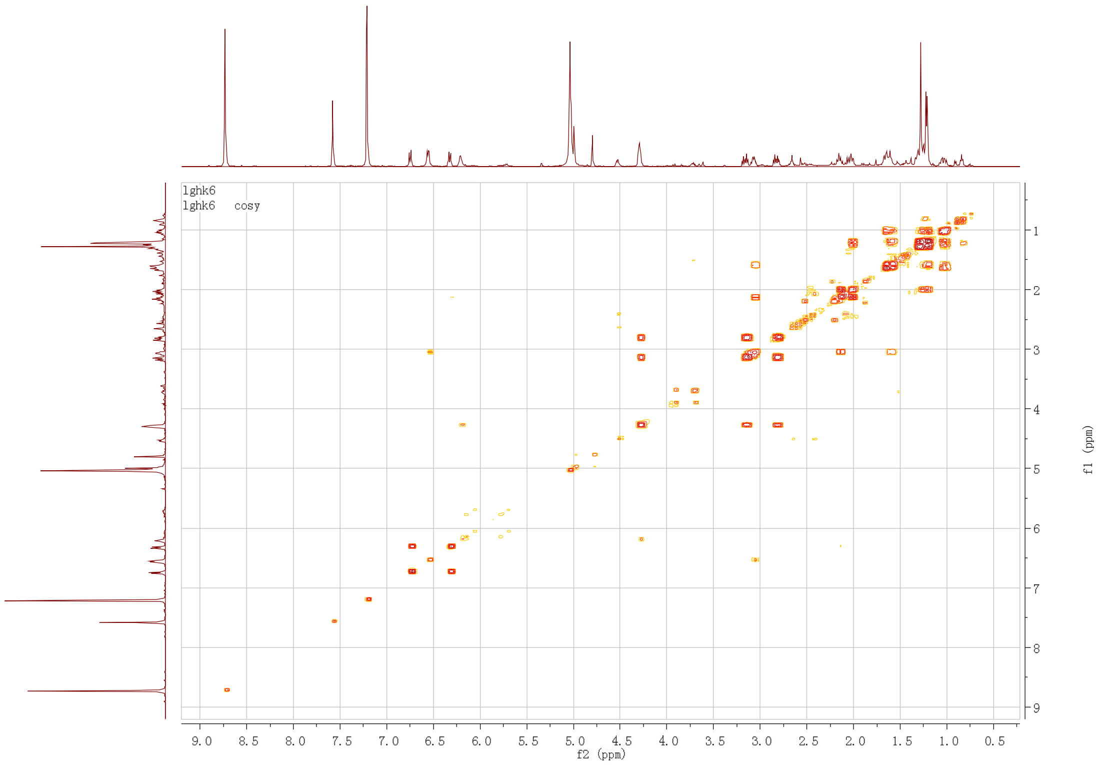

Figure 34S. ROESY of craterellone E (**5**).

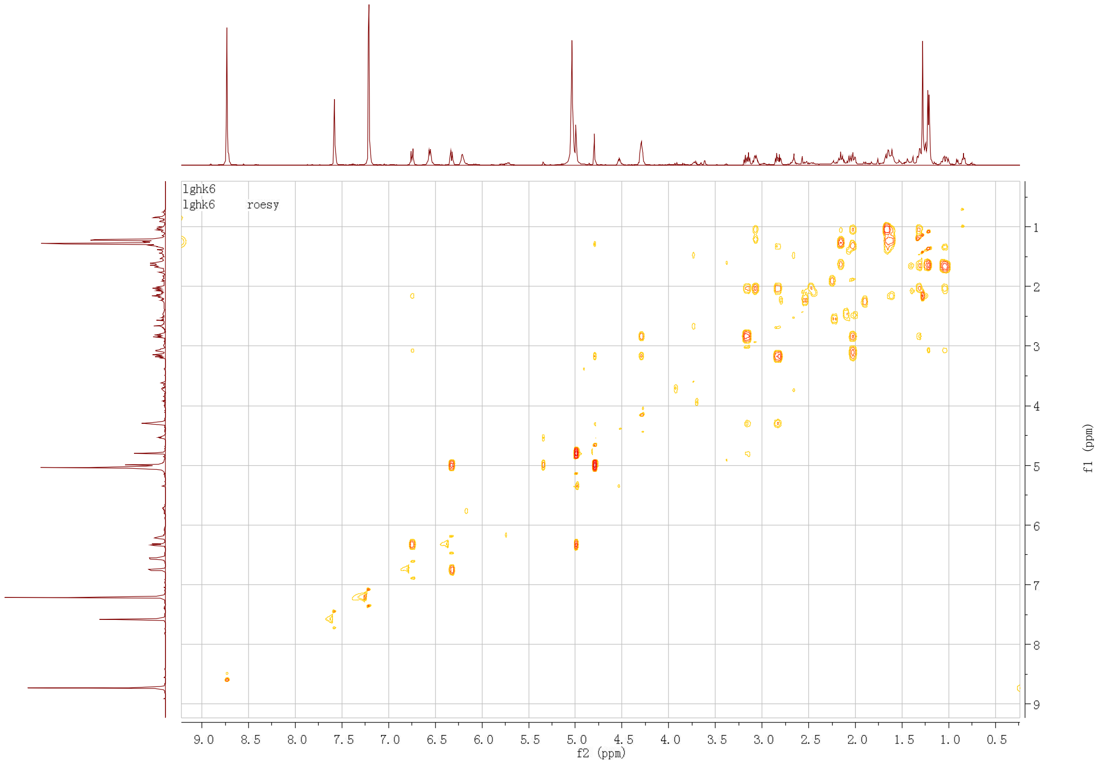

Figure 35S. HRESIMS of craterellone E (**5**).

Acq. Date: Wednesday, January 11, 2012 Acq. Time: 09:59

Sample Name: 120112ESIA Lghk6

Elemental composition calculator

Target m/z: -287.1621 amu
Tolerance: -10.0000 ppm
Result type: Elemental
Max num of results: 1000
Min DBE: -10.0000 Max DBE: +60.0000
Electron state: OddAndEven
Num of charges: 0
Add water: N/A
Add proton: N/A
File Name: 120112ESIA Lghk6.wiff

	Elements	Min Number	Max Number
1	2H	0	0
2	Br	0	0
3	C	0	200
4	Cl	0	0
5	F	0	0
6	H	0	400
7	I	0	0
8	K	0	0
9	N	0	0
10	Na	1	1
11	O	1	5
